# Neuronal Cx3cr1 Deficiency Protects against Amyloid β-Induced Neurotoxicity

**DOI:** 10.1371/journal.pone.0127730

**Published:** 2015-06-03

**Authors:** Jenny Dworzak, Benoît Renvoisé, Johnny Habchi, Emma V. Yates, Christophe Combadière, Tuomas P. Knowles, Christopher M. Dobson, Craig Blackstone, Ole Paulsen, Philip M. Murphy

**Affiliations:** 1 Laboratory of Molecular Immunology, National Institute of Allergy and Infectious Disease, National Institutes of Health, Bethesda, Maryland, United States of America; 2 Neuronal Oscillations Group, Department of Physiology, Development, and Neuroscience, University of Cambridge, Cambridge, United Kingdom; 3 Cell Biology Section, Neurogenetics Branch, National Institute of Neurological Disorders and Stroke, National Institutes of Health, Bethesda, Maryland, United States of America; 4 Department of Chemistry, University of Cambridge, Cambridge, United Kingdom; 5 Centre d'Immunologie et des Maladies Infectieuses-Paris, Institut National de la Santé et de la Recherche Médicale, Paris, France; National Center for Geriatrics and Gerontology, JAPAN

## Abstract

Cx3cr1, the receptor for the chemokine Cx3cl1 (fractalkine), has been implicated in the progression and severity of Alzheimer’s disease-like pathology in mice, but the underlying mechanisms remain unclear. A complicating factor is that Cx3cr1 has been demonstrated in both neurons and microglia. Here, we have dissected the differences between neuronal and microglial Cx3cr1, specifically by comparing direct amyloid-β-induced toxicity in cultured, mature, microglia-depleted hippocampal neurons from wild-type and *Cx3cr1^-/-^* mice. Wild-type neurons expressed both Cx3cl1 and Cx3cr1 and released Cx3cl1 in response to amyloid-β. Knockout of neuronal *Cx3cr1* abated amyloid-β-induced lactate dehydrogenase release. Furthermore, amyloid-β differentially induced depression of pre- and postsynaptic components of miniature excitatory postsynaptic currents, in a peptide conformation-dependent manner. Knockout of neuronal *Cx3cr1* abated effects of both amyloid-β conformational states, which were differentiable by aggregation kinetics and peptide morphology. We obtained similar results after both acute and chronic treatment of cultured neurons with the Cx3cr1 antagonist F1. Thus, neuronal *Cx3cr1* may impact Alzheimer’s disease-like pathology by modulating conformational state-dependent amyloid-β-induced synaptotoxicity.

## Introduction

Alzheimer's disease (AD) is a neurodegenerative disorder characterized clinically by progressive cognitive dysfunction. Pathologic hallmarks include senile plaques of aggregated amyloid-β (Aβ) and neurofibrillary tangles of hyperphosphorylated tau, coupled with dysfunctional and degenerating neurons [1]. In mouse models, Aβ evokes behavioral deficits, synaptic dysfunction, and neurotoxicity, either directly by interacting with neurons [2] or indirectly by activating microglia [3].

The G protein-coupled chemokine receptor Cx3cr1 affects Aβ neurotoxicity; however, mouse studies have been inconclusive, demonstrating both potentiation and amelioration of Aβ effects by Cx3cr1 depending on the specific model used [4–10]. This apparent conflict could result from differential cellular and regional expression of this receptor in the brain. Cx3cl1, the only known Cx3cr1 ligand, is expressed selectively on neurons in the brain and is widely thought to suppress inflammatory responses via paracrine activation of microglial Cx3cr1 [11]; consistent with this, a Cx3cr1-gfp reporter mouse has been reported to express gfp only in microglia in mouse cortex *in vivo* [4]. Nevertheless, using other methods, functional expression of Cx3cr1 has been reported in both human and rodent neurons [11–20]. Since both neurons and microglia appear to express Cx3cr1 in some contexts, defining cell-type specific roles of the receptor in affecting Aβ toxicity may provide new insights into how the receptor modulates AD-like pathology.

Previous studies have shown that excitotoxic stimuli, including Aβ administration, potentiate Cx3cr1 signaling; however, downstream effects of receptor activation are confounding. While activating *microglial* Cx3cr1 has been reported to limit neurotoxic proinflammatory mediators, suppression of Cx3cl1 signaling ameliorates neurotoxicity [7, 10, 21, 22].

Because synapse loss is closely correlated with AD symptoms [23],Aβ effects on synaptic transmission are of particular interest for AD pathogenesis. Both Aβ and neuronal Cx3cr1 suppress synaptic transmission by internalization of α-amino-3-hydroxy-5-methyl-4-isoxazolepropionic acid receptors (AMPARs) [12, 19, 24–32]. While it is known that peptide concentration and degrees of peptide aggregation influence Aβ toxicity, little is known regarding conformation-specific deficits in synaptic transmission. It is generally accepted that pre-fibrillar intermediates of Aβ, rather than fully fibrilized forms, mediate the synaptotoxic effects of the peptide; however, only a few studies indicate a peptide concentration-dependent predilection for induction of deficits in pre- or post-synaptic parameters of synaptic function [29, 33–37]. Because peptide aggregation kinetics critically depend on initial peptide concentration, it is difficult to dissect conformation-dependent effects from concentration-dependent effects of Aβ in these prior studies [38, 39].

In the present study, we have characterized specific ranges of peptide conformational states that selectively induce pre- or postsynaptic transmission deficits in cultured neurons. We also used mice with a genetic deletion of *Cx3cr1* (*Cx3cr1* ^-/-^) to analyze effects of neuronal Cx3cr1 deficiency on these measures of Aβ toxicity.

## Materials and Methods

### Primary neuronal and microglial cultures

*Cx3cr1* ^gfp/gfp^ (*Cx3cr1* knockout) mice were purchased from Jackson Laboratories (Bar Harbor, Maine) as strain B6.129P-Cx3cr1tm1Litt/J (http://jaxmice.jax.org/strain/005582.html). Chimeric mice were backcrossed to C57Bl/6 for ten generations before being made homozygous and bred to introduce the CD45.2 (Ly5.2 or Ptprcb) allele present in C57Bl/6^wt/wt^ mice, which were used as controls. Control cultures were prepared from C57Bl/6^wt/wt^ mice from Jackson Laboratories (http://jaxmice.jax.org/strain/000664.html). Both the NIAID Animal Care and Use Committee and British Home Office specifically approved all experiments conducted in this study and provided the animal study protocol and project license, under which all experiments were conducted, respectively.

### Neuronal cultures

**Fig 1:**
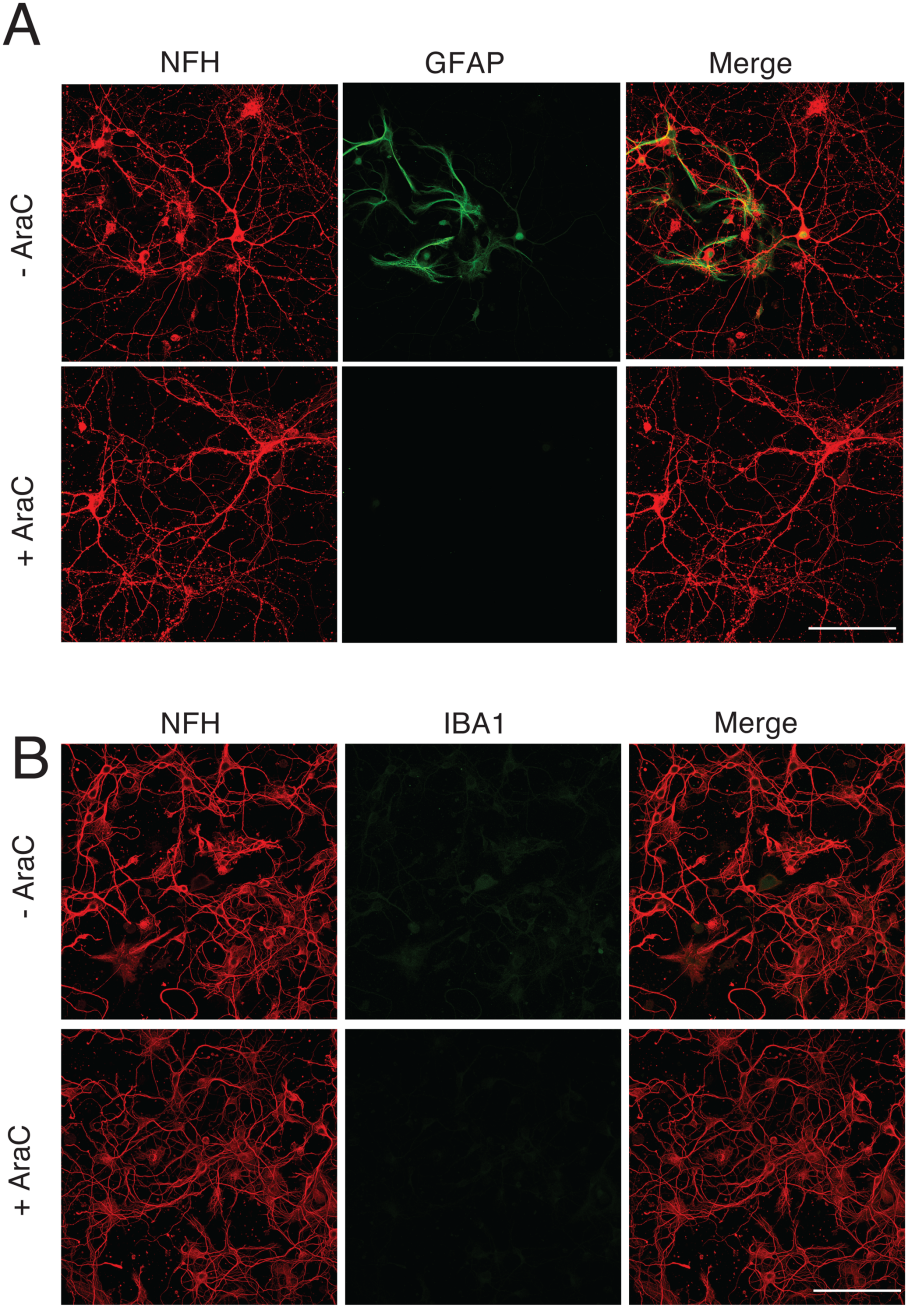
Preparation and maintenance of mature hippocampal cultures results in glial depletion at DIV21. Hippocampal primary neurons prepared from C57Bl/6 mice on P0 using a densitometric gradient centrifugation protocol were treated with 2 μM cytosine arabinoside (AraC) on DIV3 to enrich for neurons and deplete microglia. Prior to treatment at DIV3, GFAP+ cell contamination (A) was 21% ± 5 and IBA1+ cell contamination (B) was < 0.003%. At DIV 21, GFAP+ cell contamination was 6% ± 0.5 and IBA1+ cell contamination was < 0.003%. Microglial depletion was confirmed by staining for Cd11b (data not shown). *n* = 3 experiments in each sample category, with 300 cells/experiment. Scale bars, 10 μm.

Primary neuronal cultures were prepared from mouse pups at P0 or P1. Complete cerebral cortices or hippocampi were dissected and dissociated with papain (PAP2) and DNase I (Worthington Biochemical, Lakewood, New Jersey) for 45 minutes at 37°C. For immunohistochemistry, cells were plated at a density of 1.0 × 10^5^/cells per cm^2^ on 18 mm glass coverslips pre-coated with 20 μg/ml poly-L-lysine at least 24 hours prior to plating. For immunoblotting, RT-PCR, and ELISA analyses of supernatants, cells were plated at 5 × 10^6^ per 9.6 cm^2^ well in 6-well Nunc multi-well plates pre-coated with 20 μg/ml poly-L-lysine at least 24 hours prior to plating. On DIV3, cells were treated with 2 μM cytosine arabinoside, an inhibitor of DNA replication, (Sigma-Aldrich, St. Louis, Missouri) for 3 days to prevent mitotic, non-neuronal cell growth. Under these conditions, Iba1^+^ microglial contamination was <<0.1% (Fig 1), confirming previous reports [2]. Cells were maintained in B27/neurobasal (NB) medium containing 2 mM glutamine, 100 U/ml penicillin, and 100 μg/ml streptomycin in a humidified atmosphere of 95% air and 5% CO_2_ at 37°C for 21 days with partial media changes every 3 days. Each experiment (*n*) consisted of the collective mixed cortical or hippocampal tissue extracted from a single litter.

### Microglial cultures

Primary microglia were cultured from P1–P10 C57Bl/6 and *Cx3cr1* ^*-/-*^ pups. After removal of cerebellum, midbrain, and hindbrain structures, whole brain extracts were mechanically dissociated and plated in 10% FBS-containing DMEM. Cells were grown at 37°C in 5% CO_2_, with partial media changes once weekly. Monocyte colony stimulating factor (MCSF)was added on DIV10. After 2 weeks of culture, flasks were gently shaken and the supernatant containing the microglia was collected. After centrifugation, microglia were plated and maintained in 10% FBS-containing DMEM until harvested for RT-PCR.

### Immunocytochemistry

Neurons were fixed with 4% formaldehyde for 30 min at 25°C; washed with PBS; permeabilized in 0.1% Triton X-100-containing PBS for 10 minutes; and blocked in 10% donkey serum-containing PBS for 30 min. Cells were incubated overnight at 4°C with primary antibodies: NFH, polyclonal, 1:1000 dilution, Covance, Princeton, New Jersey; GFP, 1:1000 dilution, Life Technologies, Grand Island, New York; CD11b, monoclonal 1:1000 dilution, Abcam, Cambridge, Massachusetts; IBA1, polyclonal, 1:500 dilution, Wako Chemicals, Richmond, Virginia) in 0.05% Triton X-100 and 1% donkey serum-containing PBS. After washing with PBS, cells were incubated with Alexa Fluor 488- and Alexa Fluor 568-conjugated secondary antibodies (1:500 dilution; Life Technologies Molecular Probes, Grand Island, NY), in 0.05% Triton X-100 and 1% donkey serum-containing PBS for 30 min at 25°C. Coverslips were then mounted using Gel/Mount (Biomeda, Foster City, California). Fluorescent images were acquired with a Zeiss Axiovert 100M laser scanning confocal microscope. Pearson’s colocalization coefficient and Mander’s coefficients for each respective channel were obtained with Image J software. AT-8 intensity was quantified by determining integrated density of the 488 channel in all images with integrated density of the 568 channel within one standard deviation of the mean integrated density value of all images in ImageJ (NIH).

### Quantitative PCR

Total RNA was purified from cultured wild-type and *Cx3cr1* ^*-/-*^ neurons and wild-type microglia at DIV21. Cells were first washed with ice-cold PBS, then placed in TRIzol (Life Technologies) reagent after washing cells with ice-cold PBS. RNA was first extracted by addition of chloroform and precipitation with methanol and then converted to cDNA using a Superscript III Supermix kit (Life Technologies Invitrogen). Real-time PCR was performed in a total reaction volume of 22 μl using 2 μl cDNA, 11.5 μl 2X TaqMan PCR Master mix, and 1.25 μl primer—probe mix. Mouse Cx3cr1 primers used for quantitative PCR were purchased from Life Technologies Applied Biosystems (Mm00438354_m1). Real-time PCR reactions were run on an Applied Biosystems 7900HT system using the standard protocol provided by Invitrogen. Calculated copies were normalized against copies of housekeeping gene GAPDH (Applied Biosystems, cat no. Mm99999915_g1).

### LDH Cytotoxicity

Aβ (1–42) cytotoxicity was quantified by analyzing LDH content in culture medium from Aβ (1–42)- and vehicle-treated neurons using a Cytotoxicity Detection Kit (Roche Life Science, Indianapolis, Indiana), according to the manufacturer's instructions. Results were expressed as a percentage of maximal LDH release in culture medium from neurons treated with 0.2% Triton X-100 for 5 minutes immediately prior to assay.

### ELISA assay

Conditioned media collected from neurons treated with Aβ (1–42) for 24 hours on DIV 21 was assayed for soluble murine Cx3cl1 using an ELISA kit (R&D Systems, Minneapolis, Minnesota), according to the manufacturer’s instructions. Optical density was determined at 490 nm. The detection limit was 25 ng/mL.

### Pharmacology

On DIV21, neural cultures were treated with 2 μM synthetic human Aβ (1–42) (Tocris, Minneapolis, Minnesota) for determination of cytotoxicity, as indicated by lactate dehydrogenase (LDH) release. This drug concentration and culture maturity are known to exhibit microglia-independent neurotoxicity [2]. Cx3cl1 cleavage was assessed after treatment with 200 nM synthetic human Aβ (1–42), a concentration determined to induce synaptic plasticity deficits but not neuronal degeneration [40, 41]. For LDH cytotoxicity and Cx3cl1 cleavage experiments, non-1,1,1,3,3,3-Hexafluoro-2-propanol (non-HFIP; Sigma-Aldrich)-solubilized, lyophilized Aβ (1–42) was equilibrated to room temperature (22–25°C) for 30 minutes, reconstituted to 4 mM in serum-free NB media, vortexed thoroughly for 30 seconds, and sonicated for 10 minutes, before dilution to final treatment concentrations in NB medium. Culture media was assessed for LDH or Cx3cl1 content 48 hours or 24 hours after Aβ (1–42) administration, respectively.

For electrophysiology experiments, DIV14-21 microglia-depleted hippocampal cultures were incubated at room temperature with 200 nM or 2 μM Aβ (1–42) for 30 minutes prior to recording and were superperfused with identical concentrations of Aβ (1–42)-containing artificial CSF at a rate of 1.5 mL/min.

For electrophysiology experiments, non-HFIP-solubilized synthetic Aβ (1–42) was equilibrated to room temperature for 30 minutes, reconstituted to 4 μM in artificial CSF (composition described under electrophysiology methods) and sonicated for 10 minutes, before dilution to final treatment concentrations in either NB medium or artificial CSF.

HFIP-solubilized Aβ (1–42) was reconstituted, monomerized, dissolved in DMSO as described previously [42], and stored in 20 μM aliquots. Single aliquots were thawed, diluted to final treatment concentrations, vortexed for 30 seconds, and incubated at 4°C for 12–16 hours prior to treatment of culture.

### Recombinant met-Aβ (1–42) and met-Aβ (1–40)

Recombinant met-Aβ (1–42) (MDAEFRHDSGYEVHHQKLVFFAEDVGSNKGAIIGLMVGGVV IA) and met-Aβ (1–40) (MDAEFRHDSGYEVHHQKLVFFAEDVGSNKGAIIGLMVGGVV) were expressed in the *E*. *coli* BL21 Gold DE3 strain (Stratagene, La Jolla, CA) and purified as described previously, with slight modifications [43]. Briefly, the purification procedure involved sonication of *E*. *coli* cells, dissolution of inclusion bodies in 8 M urea, and ion exchange in batch mode on diethylaminoethyl cellulose resin. Eluates were analyzed using SDS-PAGE for the presence of the desired protein product. The fractions containing the recombinant protein were combined, frozen using liquid nitrogen, and lyophilized. Solutions of monomeric peptides were prepared by dissolving lyophilized peptide in 6 M GnHCl. Monomeric forms were purified from potential oligomeric species and salt using a Superdex 75 10 ⁄ 300 GL column (GE Healthcare, Buckinghamshire, UK) at a flow rate of 0.7 mL/min, and were eluted in either a 50 mM ammonium acetate buffer of sodium phosphate buffer, pH 8.5. The monomeric fractions were then lyophilized and stored as identical 0.1 mg aliquots at −80°C for further experiments. Each aliquot was only thawed and reconstituted once. Post monomer purification, oligomeric species of met-Aβ (1–42) and met-Aβ (1–40) were prepared for culture treatment as described above with and without solubilization in HFIP.

### Amino terminus-modified Cx3cr1 ligand F1

The Cx3cr1 antagonist F1 produced and characterized as described previously [44] was reconstituted in PBS at a stock concentration of 100 μM. Cultures were treated at a working concentration of 1 μM on DIV3 of culture or concurrently to treatment with met-Aβ (1–42).

### Aβ aggregation analysis by Thioflavin T (ThT) fluorescence

For aggregation kinetic analysis, 200 nM and 2 μM synthetic and recombinant A*β*, respectively, with and without HFIP solubilisation, were prepared as described above. Samples were supplemented with 20 *μ*M ThT from a 1 mM stock in half-area, black polystyrene, clear-bottomed, PEG-coated 96-well plates (Corning 3881). Experiments were performed in artificial CSF under quiescent conditions at room temperature (22-25°C). Fluorescence spectra of 20 *μ*M ThT (obtained from 1 mM stock) in separate solutions (80 *μ*L/sample) containing different A*β* preparations were measured with an Optima Fluostar plate reader (BMG Labtech, Ortenburg, Germany) (constant excitation wavelength 440 nm, emission 480 nm) for a period of 22 hours. Background fluorescence spectra obtained from 20 *μ*M ThT in plain artificial CSF was subtracted, and resultant fluorescent measurements were plotted in Graphpad Prism (Version 6). All reported fluorescence values are an average of triplicate repeats per sample. Half-times of sigmoidal aggregation curves to maximal ThT fluorescence were determined by obtaining polymer mass, accounting for monomer-dependent secondary nucleation, with the following equation, as described previously [39].

### Aβ compositional analysis by atomic force microscopy (AFM)

Samples were deposited onto freshly cleaved mica surfaces and allowed to dry for 30 minutes. At least two replicates of each condition were prepared. Samples were then washed with Milli-Q water, dried with nitrogen, and stored at 4°C. Between 3 and 7 AFM images were collected from each sample condition.

AFM images were acquired using a VEECO Dimension 3100 atomic force microscope (Brucker) and JPK Nanowizard software (Cambridge, UK). The instrument was operated in tapping mode in air using n-type silicon cantilevers with resonant frequencies between 65 and 130 kHz and force constants between 0.6 and 2 N/m. Tip (MikroMasch, Wetzlar, Germany) radii, heights, and cone angles were 8 nm, between 12 and 18 μm, and 40 degrees, respectively. Bulk probe sensitivity was between 0.01 and 0.025 Ω/cm. Probe mat Images were collected at a scan rate of 0.5 Hz, and with resolution varying between approximately 0.5 and 24 nm/pixel.

Image preprocessing was conducted using Gwyddion software and included background leveling with a fourth order polynomial correction, and correcting horizontal lines by matching height median, and horizontal scars, if necessary. Image features were identified in Gwyddion. A thresholding algorithm was used for the recombinant samples with and without HFIP with height thresholds of between 8.5 and 9.5% of maximal values. The synthetic HFIP and non-HFIP samples had complex and feature-rich backgrounds, so in these cases a watershed algorithm was used for enhanced segmentation. Image specific parameters were applied for optimal extraction of features over background. In each case, several thousand image features were considered. The maximum value of each image feature was measured One or several Gaussian distributions were fit to these data in Matlab and the mean and standard deviation of the peak(s) were extracted.

### Electrophysiology

#### Intracellular and extracellular recording solutions

Borosilicate glass recording pipettes of 3–5 MΩ resistance were filled with intracellular solution containing 120 mM CsCH_3_SO_3_, 20 mM CsCl, 0.2 mM EGTA, 10 mM HEPES, 10 mM QX-314, 0.3 mM GTP, and 4 mM Mg-ATP with osmolarity of 285–295 mOsm/L and pH adjusted to 7.25–7.30 using CsOH. Bath artificial CSF contained 145 mM NaCl, 3 mM KCl, 15 mM HEPES, 2 mM CaCl_2_, 10 mM glucose, 2 mM MgCl_2,_ with osmolarity of 295–305 mOsm/L and pH of 7.40–7.45 adjusted by NaOH. Tetrodotoxin (TTX; 1 μM) and gabazine (GBZ; 1 μM) were added to the bath solution for isolation of AMPAR-mediated mEPSCs. Patched cells were allowed 10 minutes of dialysis and equilibration after perforation and were recorded for 5–10 minutes at a holding potential of -80 mV. Isolated mEPSCs at -80 mV were abolished by 10 μM CNQX.

### mEPSC recording and analysis parameters

Whole-cell mEPSC recordings were obtained using an Axon Multiclamp 700B amplifier (Molecular Devices, Sunnyvale, CA). Recordings were low pass-filtered at 2 kHz and acquired at 5 kHz using an Instrutech ITC-16 acquisition board (Instrutech, Port Washington, NY) and custom-made acquisition protocols programmed in Igor Pro software (WaveMetrics, Lake Oswego, OR). All experiments were performed in voltage clamp mode at a holding potential of −80 mV. GABAergic currents were assessed at 0 mV and were completely blocked under the described recording conditions. Recordings were discarded if leak current was greater than 200 pA, input resistance was greater than 100 MΩ, series resistance was greater than 30 MΩ, or if series resistance fluctuated more than 15%.

mEPSC detection and analysis were conducted by a semi-automated method using custom protocols programmed in Igor Pro (Wavemetrics). Analysis was limited to recordings with a minimum of 50 events in each one-minute interval analyzed and averaged. We first calculated the first derivative of the raw data trace with respect to time; events were defined as negative amplitude fluctuations of 5–100 pA with monotonic rise slopes of the first derivative of the tracing at least twice that of baseline noise and exponential decay time constants less than 25 ms. Frequency, peak amplitude, and decay times were averaged over at least 3 recordings per coverslip. At least 3 coverslips were averaged per experimental n.

### Statistical Analysis

Unless otherwise noted, all data plots are mean ± SEM. We used Kolmogorov-Smirnov testing to determine normal distribution of data; for non-normal distributed data or for data whose sample size was insufficient to confirm normal distribution, we applied the non-parametric Mann-Whitney test. For multi-factor interaction testing, one- and two-way ANOVAs were used, as applicable. Values are considered statistically significant if *P* < 0.05 or, in the cases of multiple-factor testing, if *P* values meet post-hoc Bonferroni-corrected thresholds for significance. All two-tailed Student *t*-tests, one-way and two-way ANOVAs, non-parametric Mann-Whitney tests were performed using Prism, version 6 (GraphPad Software).

## Results

### Cultured mature microglial-deficient hippocampal neurons express Cx3cr1

Because neuronal Cx3cr1 expression in mice is controversial [11–16] and has not yet been investigated in our system, we first assayed mature microglia-depleted hippocampal cultures from postnatal *Cx3cr1* ^*+/gfp*^ heterozygous mice for Cx3cr1 expression. Microglial depletion was attained using a rigorous densitometric segregating centrifugation protocol (see Materials and Methods) and ensured for the lifespan of the culture by treatment with 2 μM cytosine arabinoside (AraC) on DIV3 (Fig 1).

**Fig 2:**
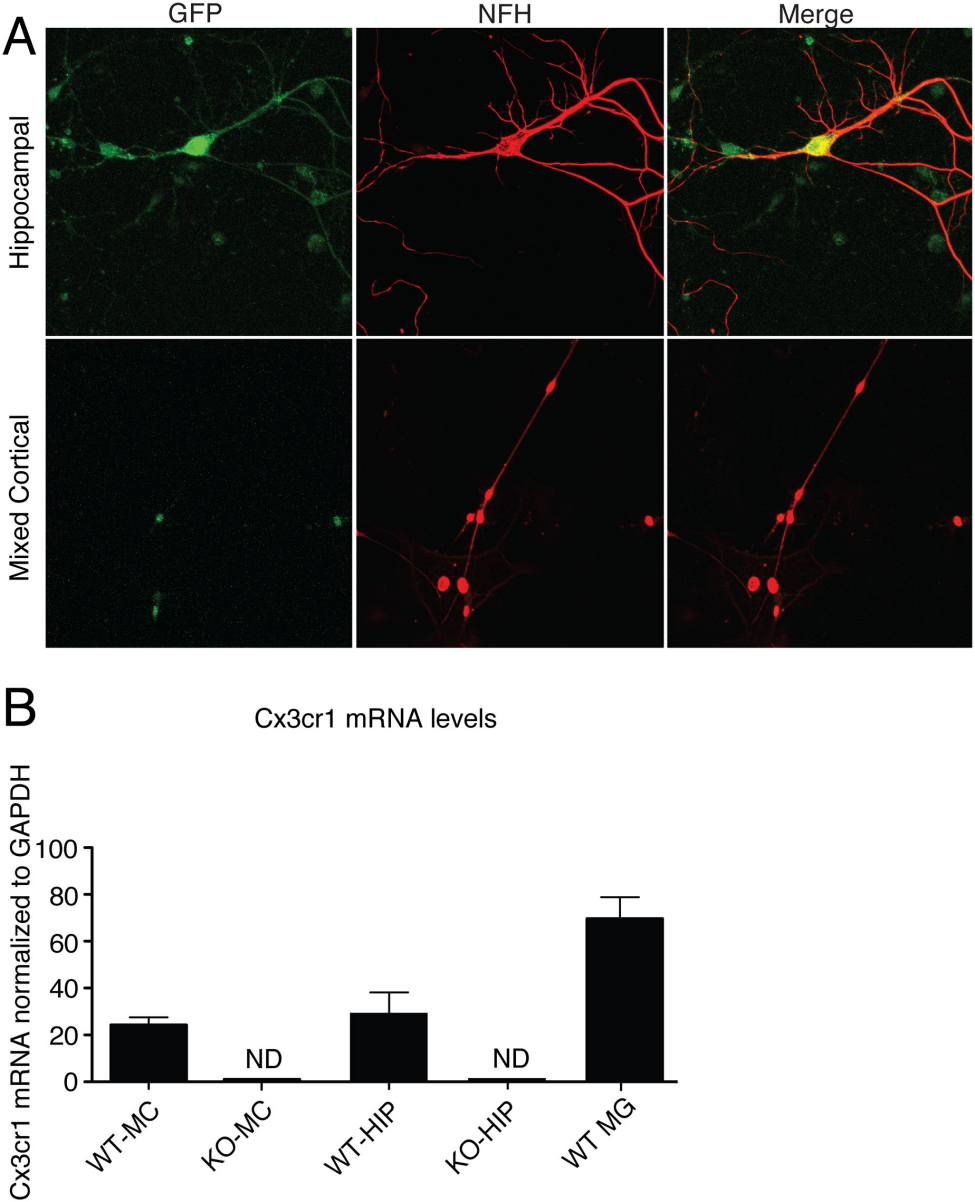
Postnatal microglia-depleted neuronal cultures express Cx3cr1 protein and mRNA. Mature mixed cortical (MC) and hippocampal (HIP) cultures prepared from postnatal Cx3cr1^+/GFP+^ mice and treated with 2 μM cytosine arabinoside (AraC) were stained with Neurofilament-H (NFH) (red) and anti-GFP (green). (A) Colocalization of Cx3cr1-GFP and NFH in hippocampal and mixed cortical neurons. Cx3cr1-GFP significantly colocalizes with NFH+ cell bodies, axons and dendrites in hippocampal culture. Pearson’s coefficient of covariance was 0.39 ± 0.03 over all high power fields. Mander’s coefficient for the red channel was 0.89 and for the green channel was 0.70. Coste’s value of significance of colocalization was 1.00. *n* = 6 in each sample category. (B) Neuronal Cx3cr1 mRNA. MC or HIP neuronal cultures were prepared from either wild type C57Bl/6 (WT) or *Cx3cr1* ^*gfp/gfp*^ (KO) mice. ND, not detected. Scale bars, 50 μm.

We found that EGFP expressed under the endogenous *Cx3cr1* promoter significantly colocalized with Neurofilament H (NFH)-positive cell bodies, axons, and dendrites in hippocampal cultures (Fig 2A). Manual cell counts of NFH- and EGFP-positive cell bodies indicated that the majority of NFH-positive neurons in hippocampal culture (91.9% ± 2.4%) were also positive above background for EGFP, a result corroborated by Cx3cr1 RNA transcript detection (Fig 2B). While mixed cortical cultures also expressed Cx3cr1 RNA, less than half of NFH-positive neurons (36% ± 7.4%) in these cultures were also positive for EGFP above background. These results indicate that NFH-positive neurons within our system of mature, microglia-deficient neuronal culture expressed *Cx3cr1* RNA and protein and that the degree of protein expression was regionally selective (*P* = .01, two-tailed Student’s *t*-test).

### *Cx3cr1* promotes Aβ(1–42)-induced neurotoxicity *in vitro*

Since microglial Cx3cr1 limits the neurotoxic inflammatory milieu [4–10, 45], we next investigated whether or not *neuronal* Cx3cr1 might, in contrast, directly potentiate Aβ (1–42) neurotoxicity in culture.

**Fig 3:**
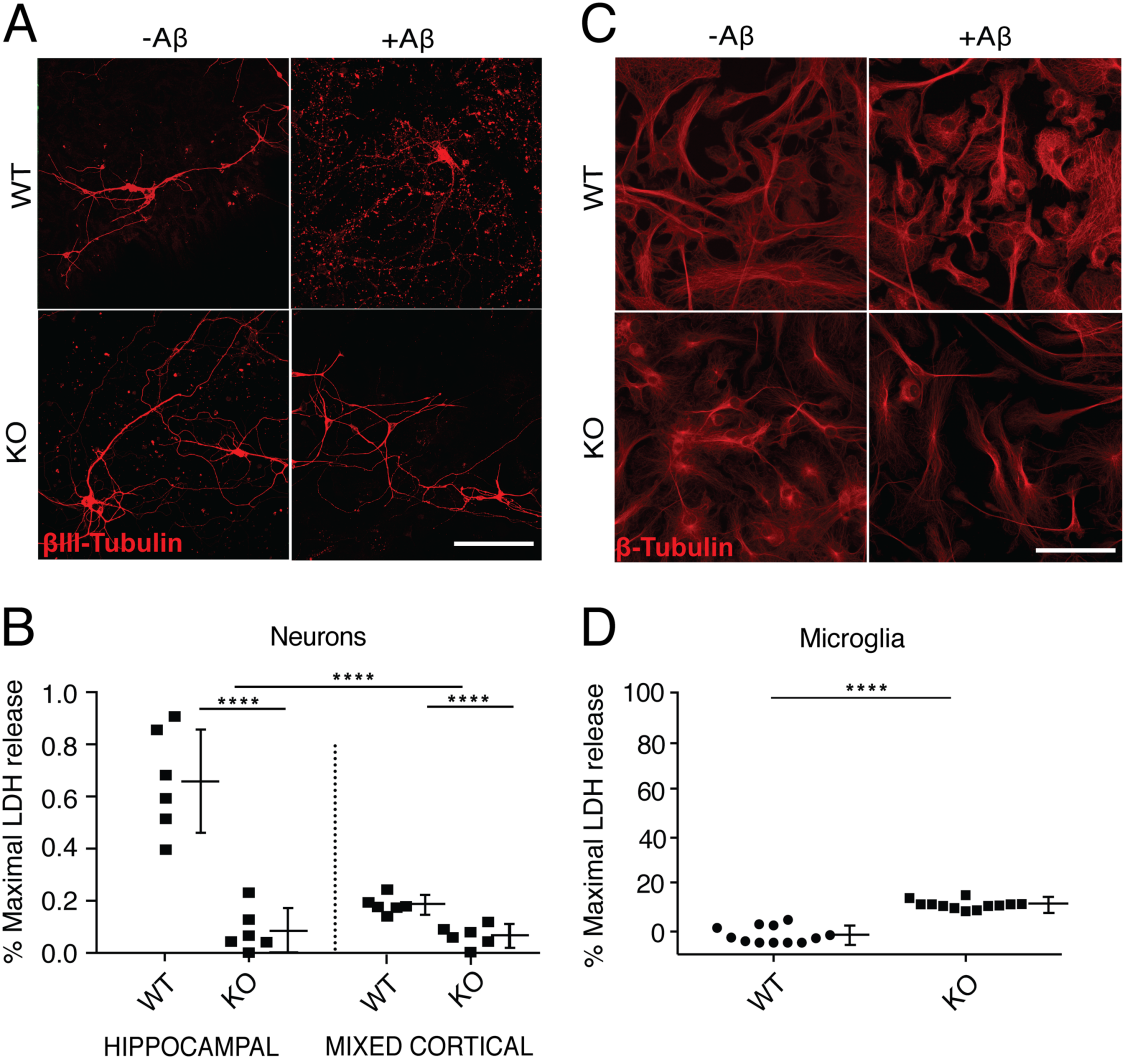
*Cx3cr1* promotes Aβ(1–42)-induced cytotoxicity, preferentially in hippocampal neurons. (A) Aβ(1–42)-induced cell loss, measured by β-tubulin staining patterns of mature microglia-depleted cultured hippocampal neurons from C57Bl/6 (WT) or *Cx3cr1* ^*-/-*^ (KO) mice. Scale bar, 10 μm. (B) Aβ(1–42)-induced cytotoxicity, quantified biochemically as % of maximal LDH release in microglial-depleted hippocampal and mixed cortical neurons. Maximum LDH release was defined using control neurons, or microglia respectively, treated with 2% Triton X-100. Results are summarized as the mean ± SEM of cumulative data from 7 separate experiments repeated in triplicate for each genotype. **** signifies *P* = 0.0001, two-way ANOVA. (C) Aβ(1–42)-induced cell loss, measured by tubulin staining patterns of pure microglia. Scale bar, 10 μm. (D) Aβ(1–42)-induced cytotoxicity, quantified biochemically as % of maximal LDH release in pure microglia cultures. Maximum LDH release was defined using control neurons, or microglia respectively, treated with 2% Triton X-100. Results are summarized as mean ± SEM of cumulative data from 7 separate experiments repeated in triplicate for each genotype. ****P = 0.0001, Mann-Whitney.

To test whether or not knockout of neuronal *Cx3cr1* abates Aβ (1–42) neurotoxicity, we treated wild-type and *Cx3cr1* ^*-/-*^ neurons from microglia-depleted mixed cortical and hippocampal cultures with 2 μM human Aβ (1–42) on DIV21, conditions previously identified to induce neurotoxicity directly in the absence of glia [2, 46]. We used LDH release and βIII-tubulin immunoreactivity (Fig 3A) to assess Aβ(1–42)-induced cytotoxicity, and found significantly reduced levels of LDH release and cell loss in cultured *Cx3cr1* ^*-/-*^ neurons compared to wild type neurons (Fig 3B).

Consistent with the neuronal Cx3cr1 expression data, the reduction in LDH release conferred by *Cx3cr1* deficiency was more pronounced in hippocampal cultures than in mixed cortical cultures. In contrast, we found that Aβ(1–42) induced significantly increased levels of LDH release and cell loss as assessed by tubulin immunoreactivity in purified microglia cultures from *Cx3cr1* ^*-/-*^ mice compared to those from wild type mice, indicating that Cx3cr1 differentially affects cellular responses to Aβ(1–42) in neurons and in microglia (Fig 3C and 3D).

### Aβ(1–42) potentiates Cx3cl1 cleavage from neurons

Cx3cl1 is a plasma membrane protein that can be cleaved to release the N-terminal chemokine domain, which signals either in a paracrine or autocrine manner through Cx3cr1. Since inflammatory cytokines and Aβ itself enhance Cx3cl1 signalling preceding cell death in various disease models [7, 10, 21, 22], we asked whether Aβ (1–42) also potentiates Cx3cl1 cleavage in microglia-depleted cultured neurons. ELISA analysis of media from wild-type neuronal cultures that had been treated with 200 nM Aβ (1–42) for 24 hours demonstrated a marked increase in soluble Cx3cl1 compared to media from vehicle-treated controls, whereas Cx3cl1 mRNA transcript levels did not differ significantly from baseline. Absolute values of Cx3cl1 concentration were 0.17 ± 0.02 ng/mL in media from vehicle-treated hippocampal neurons and 1.29 ± 0.45 ng/mL in media from Aβ (1–42)-treated hippocampal neurons (*P* = .0001, Mann-Whitney). Data analyzed consisted of cumulative data from four different experiments repeated in triplicate for each condition. This finding suggests that Aβ (1–42) potentiates Cx3cl1 release from neurons prior to cell death.

### Distinct aggregation states of Aβ(1–42) induce pre- or postsynaptic toxicity selectively, both of which are dependent on neuronal Cx3cr1

Since cognitive dysfunction correlates more closely with synapse loss than with cell loss in humans and because synaptic dysfunction precedes degeneration in AD animal models [23], we investigated the effects of Aβ (1–42) treatment on AMPAR-dependent miniature excitatory postsynaptic currents (mEPSCs) in mature microglia-depleted hippocampal cultures. AMPAR-dependent mEPSCs, representative of network stabilization, are perturbed in AD mouse models [24–26, 28–30, 47]; mEPSC frequency primarily reflects presynaptic release probability, while mEPSC amplitude primarily reflects postsynaptic mechanisms of receptor trafficking and sensitization.

Because synaptotoxicity appears to be critically dependent on precise conformational states of Aβ and because oligomeric intermediates of Aβ (1–42) are considered the most potent synaptotoxic species [41], we characterized the physico-chemical properties of several Aβ preparations, known to induce either cytotoxicity or acute synaptic plasticity deficits, by assessing the aggregation kinetics of fibril formation for each preparation under experimental recording conditions.

**Fig 4:**
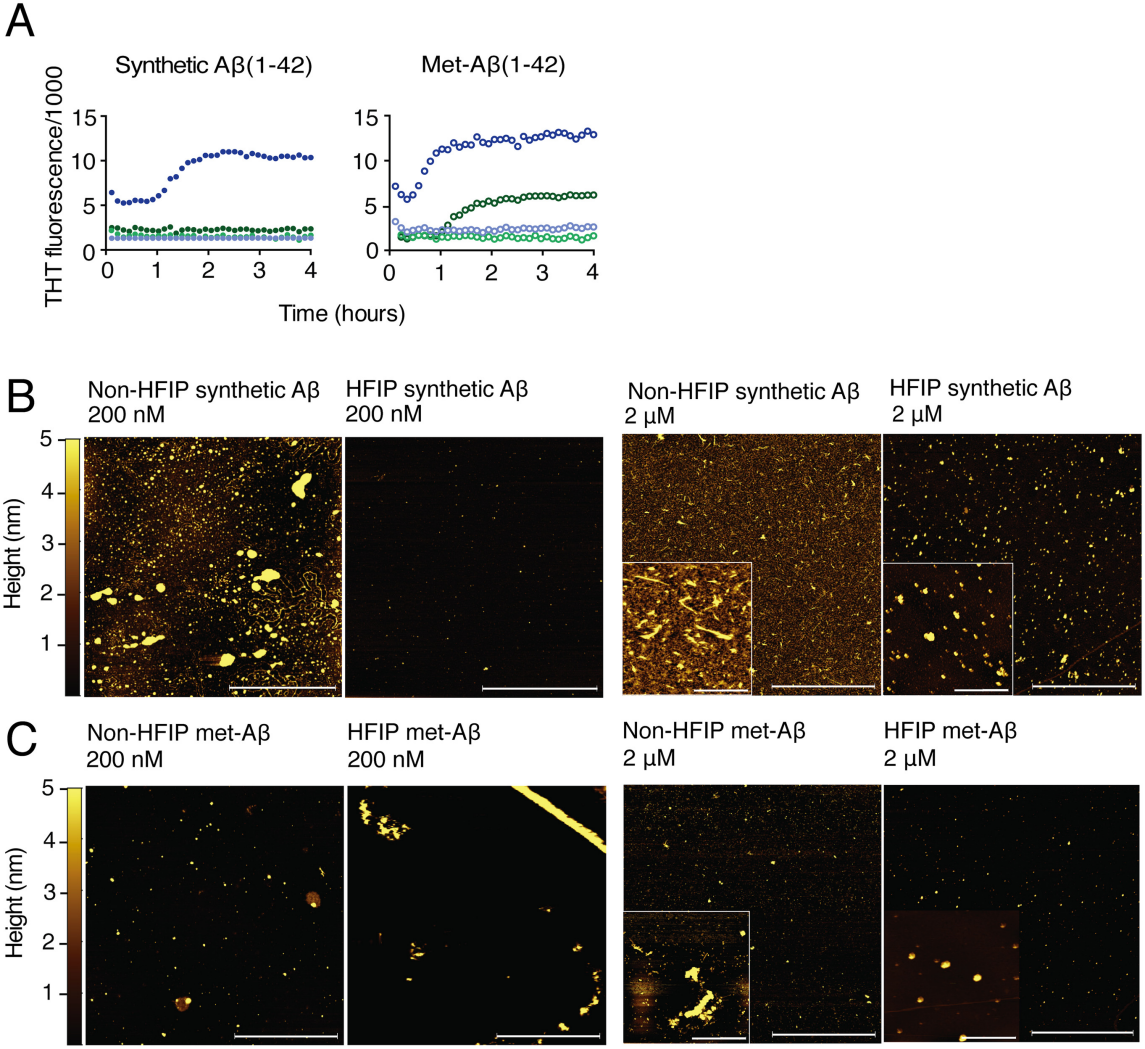
Aβ (1–42) aggregation kinetics are dictated by peptide source, concentration, and preparation protocol. (A) Experimental aggregation kinetics of synthetic and recombinant Aβ(1–42) as measured by ThT fluorescence intensity. 2 μM synthetic Aβ(1–42): non-HFIP (solid dark blue circles), HFIP Aβ (1–42) (solid dark green circles); 2 μM recombinant met-Aβ (1–42): non-HFIP (open dark blue circles), HFIP (open dark green circles). 200 nM synthetic Aβ(1–42): non-HFIP (solid light blue circles), HFIP (solid light green circles); 200 nM recombinant met-Aβ (1–42): non-HFIP (open light blue circles), HFIP (open light green circles). Graphed data represent average of 3 experiments conducted in triplicate. See Table 1 for half-times and errors. (B) AFM images of non-HFIP and HFIP-monomerized synthetic Aβ(1–42). Panel scale bars, 4 μm. Inset scale bars, 400 nm. (C) AFM images of non-HFIP and HFIP-monomerized recombinant met-Aβ(1–42). Panel scale bars, 4 μm. Inset scale bars, 400 nm.

Synthetic Aβ (1–42) was dissolved in aCSF directly, as done previously to induce Aβ synaptotoxicity [40], or dissolved in 1,1,1,3,3,3-Hexafluoro-2-propanol (HFIP) and solubilized in DMSO to form amyloid-derived diffusible ligands (ADDLs), a species closely linked to Aβ toxicity [34, 48, 49]. Each Aβ (1–42) preparation was added to cultured microglia-depleted hippocampal neurons for 30–45 minutes at a concentration of 200 nM, the minimum concentration found to induce synaptic effects [50], or 2 μM, at which we previously found Aβ (1–42)-induced neurotoxicity after 48 hours in microglia-depleted cultures. We found that only 2 μM non-HFIP-solubilized-Aβ (1–42) (non-HFIP Aβ) exhibited rapid sigmoidal kinetics, attaining complete fibril formation within 2 hours. Conversely, 200 nM preparations and 2 μM HFIP-solubilized-Aβ (1–42) (HFIP Aβ) did not aggregate during the recording interval (Fig 4A). Because toxicity-modifying impurities have been reported in synthetic Aβ [51], we conducted similar experiments with purified recombinant met-Aβ (1–42) and obtained analogous results, with the notable exception of 2 μM HFIP-met-Aβ (1–42), which also rapidly aggregated within the experimental window. Table 1 lists comparative half-times to maximal ThT fluorescence.

**Table 1:** Aggregation kinetics of synthetic and recombinant Aβ (1–42) and Aβ (1–40).

Initial peptide	Peptide preparation	Initial peptide concentration	T_1/2_ (hrs)^1^ ± standard deviation (SD)
Synthetic Aβ (1–42)	Non-HFIP	200 nM	-
Synthetic Aβ (1–42)	Non-HFIP	2 μM	1.22 ± 0.06
Synthetic Aβ (1–42)	HFIP	200 nM	-
Synthetic Aβ (1–42)	HFIP	2 μM	11.29 ± 0.2
Rec.^2^ met-Aβ (1–42)	Non-HFIP	200 nM	-
Rec. met-Aβ (1–42)	Non-HFIP	2 μM	0.76 ± 0.7
Rec. met-Aβ (1–42)	HFIP	200 nM	-
Rec. met-Aβ (1–42)	HFIP	2 μM	1.16 ± 0.08
Rec. met-Aβ (1–40)	Non-HFIP	200 nM	-
Rec. met-Aβ (1–40)	Non-HFIP	2 μM	16.1 ± 0.09^3^
Rec. met-Aβ (1–40)	HFIP	200 nM	-
Rec. met-Aβ (1–40)	HFIP	2 μM	-
Rec. met-Aβ (1–40)	IPP^3^-NaP buffer^4^	200 nM	-
Rec. met-Aβ (1–40)	IPP-NaP buffer	2 μM	-
Rec. met-Aβ (1–40)	IPP-aCSF	200 nM	-
Rec. met-Aβ (1–40)	IPP-aCSF	2 μM	16.1 ± 0.01^5^

^1^ T_1/2_ beyond experimental window of 22 hours were not assessed

^2^ Rec. met-Aβ refers to the recombinant peptide (See methods for full description).

^3^ IPP refers to peptide addition immediately post-purification without any pre-treatment preparation

^4^ All samples were aggregated in aCSF per materials and methods except for those denoted as prepared in sodium-phosphate (NaP) buffer

^5^ Sonication of Aβ (1–40) for 10 minutes in aCSF prior to addition to neuronal culture does not significantly change aggregation kinetics of the Aβ (1–40) monomer directly after purification

Because aggregation is critically dependent on properties of seeding species [38], we performed AFM to identify the initial species corresponding to these distinct aggregation profiles (Fig 4B) and found that 2 μM HFIP Aβ (1–42) (the only 2 μM sample that did not aggregate during the experimental window) demonstrated two distinct populations of spherical oligomers with *z*-height values of 0.5–1.0 nm or 14–20 nm, either of which may have precluded sigmoidal aggregation during the experimental window. All other 2 μM preparations were comprised primarily of polydisperse spherical oligomers without evidence of the two distinct populations observed in the 2 μM HFIP synthetic Aβ (1–42) preparation described above. Specifically, relative to its comparable synthetic preparation, HFIP 2 μM recombinant met-Aβ (1–42) demonstrated a more homogenous distribution of spherical oligomers between 3 and 6 nm and no significant spherical oligomer populations with z-heights of 0.5–1.0 nm or 14–20 nm (Fig 4C).

**Fig 5:**
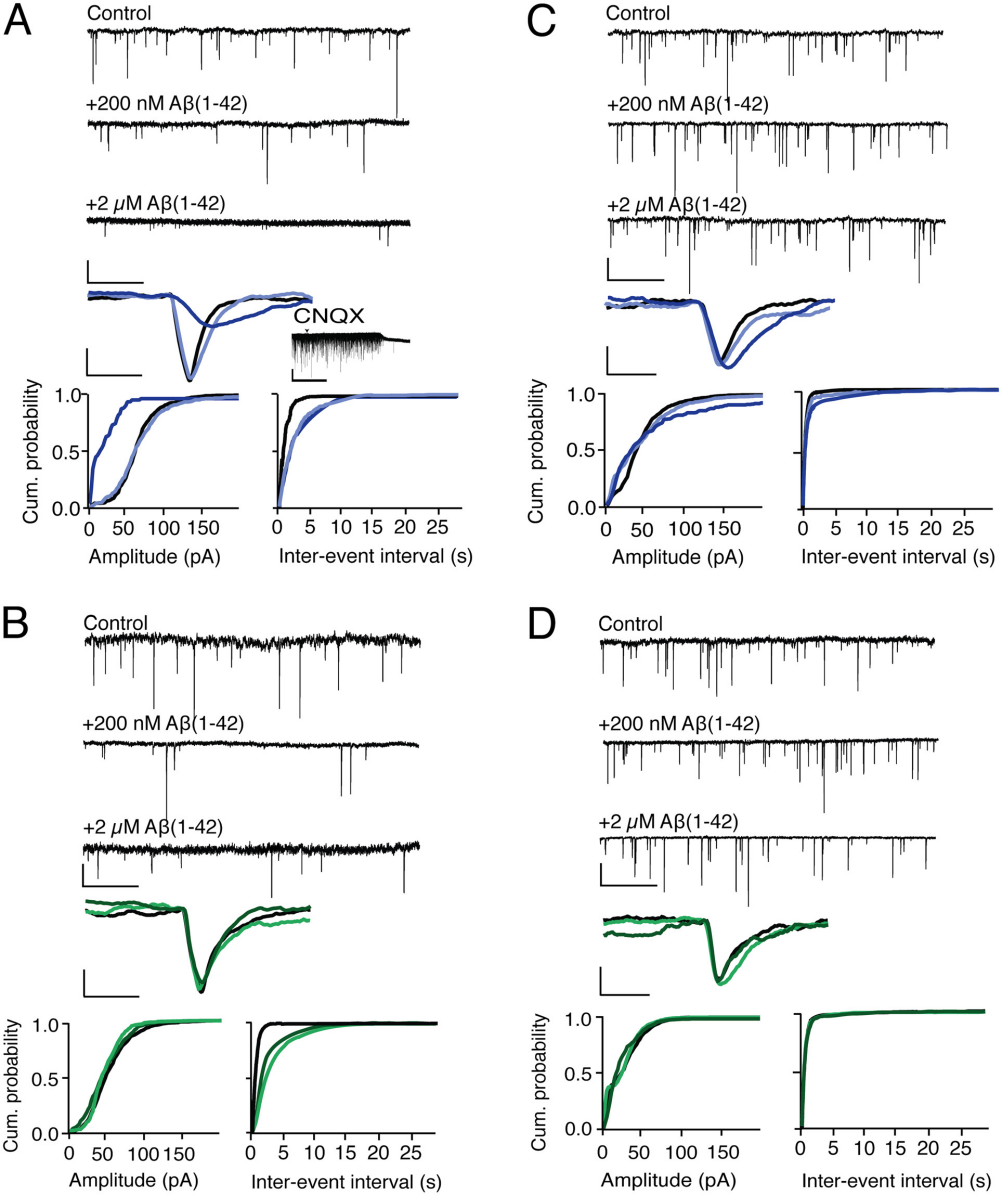
Aggregation state of synthetic Aβ(1–42) determines its ability to depress pre- or postsynaptic components of mEPSCs, and *Cx3cr1* ^*-/-*^ hippocampal neurons are resistant to both effects. Pre- and post-synaptic components of mEPSCs are measured as mEPSC frequency and amplitude, respectively. (A inset) Recorded mEPSCs were abolished by 10 μM CNQX Arrow indicates time of CNQX administration. (A) Non-HFIP synthetic Aβ(1–42) in wild-type hippocampal neurons. (B) HFIP synthetic Aβ(1–42) in wild-type hippocampal neurons. (C) Non-HFIP synthetic Aβ(1–42) in *Cx3cr1* ^*-/-*^ hippocampal neurons. (D) HFIP synthetic Aβ(1–42) in *Cx3cr1* ^*-/-*^ hippocampal neurons. For (A) through (D): the top three tracings are representative tracings of multiple mEPSCs recorded in the conditions indicated above each tracing. The single mEPSC is a representative tracing of the average amplitude, rise- and decay-time of recordings from 6 independent experiments, each of which averaged recordings from 3 separate coverslips. Cumulative probabilities represent averages of all 6 experiments. For the bottom three graphs: control, solid black line; 200 nM Aβ(1–42), light blue line (A) and (D), and light green line (B) and (D); 2 μM Aβ(1–42), dark blue line (A) and (C) and dark green line (B) and (D). For (A)–(D), the scale bars for multiple mEPSC tracings: vertical, 20 pA and horizontal, 1 s; for multiple mEPSCs treated with CNQX: vertical, 50 pA and horizontal, 1 min; and for single mEPSC tracings: vertical, 20 pA and horizontal, 6 ms. See Tables 2 and 3 for all parameters and *P* values. See Fig 6E and 6F for summary of comparison between parameters of wild-type and Cx3cr1-/- hippocampal neurons.

**Fig 6:**
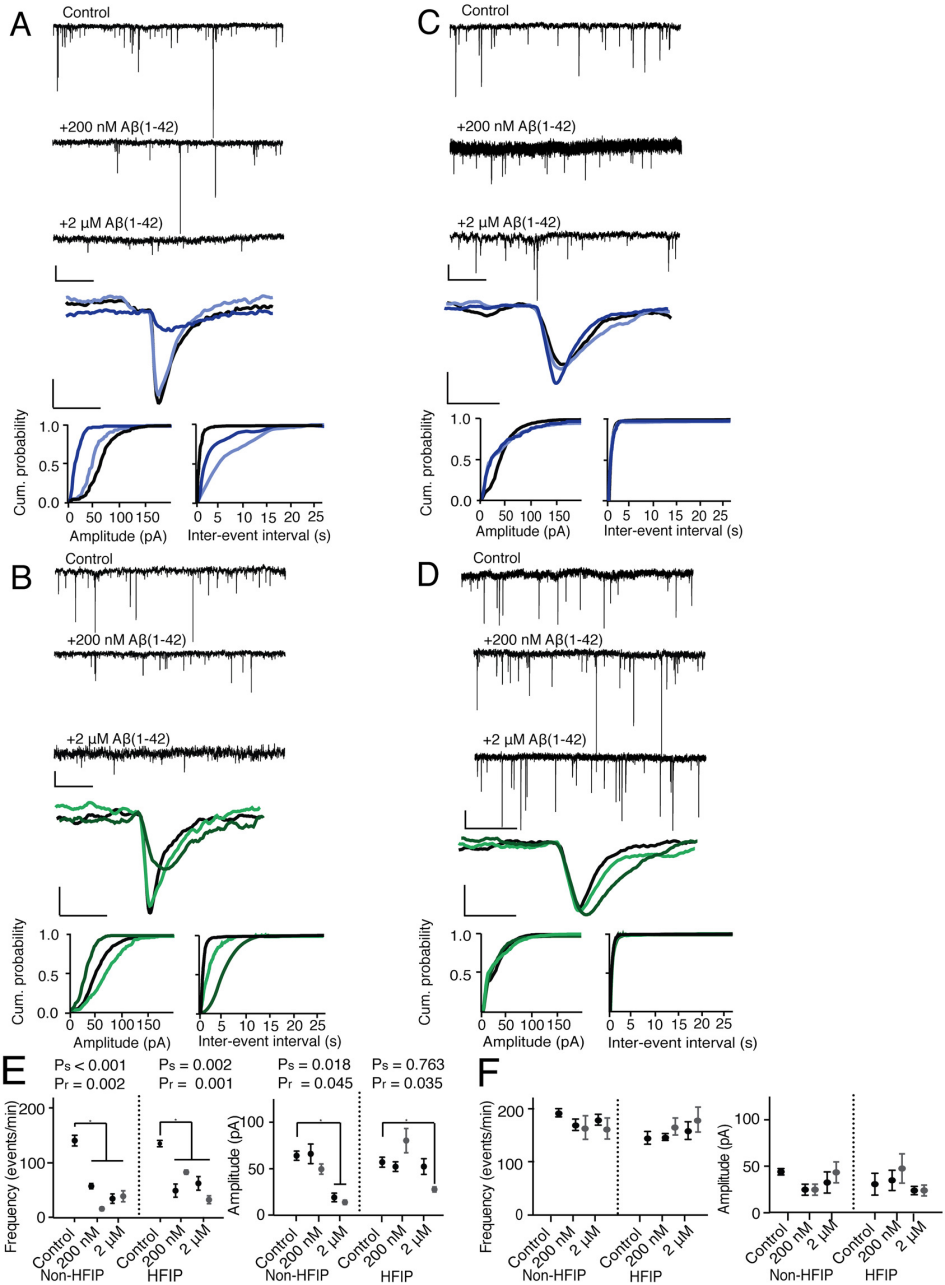
Aggregation state of recombinant met-Aβ(1–42) determines its ability to depress pre- or postsynaptic components of mEPSCs, and *Cx3cr1* ^*-/-*^ hippocampal neurons are resistant to both effects. (A) Non-HFIP recombinant met-Aβ(1–42) in wild-type hippocampal neurons. (B) HFIP recombinant met-Aβ(1–42) in wild-type hippocampal neurons. (C) Non-HFIP recombinant met-Aβ(1–42) in *Cx3cr1* ^*-/-*^ hippocampal neurons. (D) HFIP recombinant met-Aβ(1–42) in *Cx3cr1* ^*-/-*^ hippocampal neurons. For (A) through (D): the top three tracings are representative tracings of multiple mEPSCs recorded in the conditions indicated above each tracing. The single mEPSC is representative tracing of the average amplitude, rise- and decay-time of recordings from 6 independent experiments, each of which averaged recordings from 3 separate coverslips. Cumulative probabilities represent averages of all 6 experiments. For the bottom three graphs: control, solid black line; 200 nM Aβ(1–42), light blue line (A) and (D) and light green line (B) and (D); 2 μM Aβ(1–42), dark blue line (A) and (C) and dark green line (B) and (D). Recorded mEPSCs were abolished by 10 μM CNQX. For (A) through (D), the scale bars for multiple mEPSC tracings are: vertical, 20 pA and horizontal, 1 s; for multiple mEPSCs treated with CNQX: vertical, 50 pA and horizontal, 1 min; and for single mEPSC tracings: vertical, 20 pA and horizontal, 6 ms. See Table 2 for all parameters and P values. (E) Summary of mEPSC amplitudes and frequencies for synthetic (solid black circles) and recombinant (solid gray circles) Aβ(1–42)-treated wild-type neurons. (F) Summary of mEPSC amplitudes and frequencies for synthetic (solid black circles) and recombinant (solid gray circles) Aβ(1–42)-treated *Cx3cr1* ^*-/-*^ neurons. Data were inspected for normality of distribution of frequency and amplitude values by the Kolmogorov-Smirnov test, analyzed for statistical significance by two-way ANOVA by genotype and treatment condition, with posthoc Bonferroni correction for multiple testing. Aβ concentrations and preparation protocols are indicated on the x-axes. Control, incubated with aCSF only. Data represent mean ± SEM of 6 independent experiments in each condition. *P* values for difference in parameters after treatment with synthetic (s) or recombinant (r) Aβ are shown. See Tables 2 and 3 for all parameters.

When synthetic Aβ (1–42) preparations were added to microglia-depleted hippocampal culture, we found that 2 μM non-HFIP Aβ (1–42) (aggregating Aβ) reduced both mean mEPSC frequency and amplitude, while 2 μM HFIP Aβ (1–42) and both 200 nM preparations (non-aggregating) decreased mean mEPSC frequency only (Fig 5A and 5B). See Table 2 for all parameters. These results suggest that Aβ(1–42) species present in both non-aggregating and aggregating preparations of Aβ selectively depress presynaptic parameters of mEPSCs, while species present during the rapid sigmoidal conformational transition of Aβ to aggregated fibrils affect both pre- and postsynaptic parameters of mEPSCs. Similarly, only aggregating preparations of recombinant met-Aβ also depressed mEPSC amplitude (Fig 6A and 6B).

**Table 2:** Aβ-induced mEPSC depression in microglia-depleted mature wild type hippocampal neurons.

Parameter		Prep.	Conc.	Value	SEM
Frequency^1^	Control	Non-HFIP		2.34	0.16
Amplitude^2^				64.07	4.96
Decay Time^3^				2.00	.21
Frequency		HFIP		2.25	0.10
Amplitude				57.10	5.37
Decay Time				3.10	.57
Frequency	Syn. Aβ (1–42)	Non-HFIP	200 nM	0.96	0.08
Amplitude				66.11	10.55
Decay Time				4.64	0.79
Frequency			2 μM	0.58	0.14
Amplitude				22.61	3.53
Decay Time				6.23	0.97
Frequency		HFIP	200 nM	0.82	0.20
Amplitude				52.36	5.24
Decay Time				3.05	.65
Frequency			2 μM	1.05	0.21
Amplitude				52.43	8.47
Decay Time				2.38	.30
Frequency	Rec. met-Aβ (1–42)	Non-HFIP	200 nM	0.27	0.06
Amplitude				49.66	5.46
Decay Time				3.64	.93
Frequency			2 μM	0.65	0.17
Amplitude				14.01	2.33
Decay Time				5.03	0.97
Frequency		HFIP	200 nM	1.34	0.06
Amplitude				80.33	13.07
Decay Time				2.76	0.37
Frequency			2 μM	0.55	0.12
Amplitude				27.85	2.76
Decay Time				10.02	2.07
Frequency	Rec. met-Aβ (1–40)	Non-HFIP	2 μM	2.19	0.31
Amplitude				58.63	13.53
Decay Time				3.07	0.32
Frequency		HFIP	2 μM	2.22	0.02
Amplitude				51.84	8.87
Decay Time				2.00	0.21
Frequency		IPP^4^	2 μM	2.06	0.14
Amplitude				41.92	9.37
Decay Time				2.88	0.19

^1^ mEPSC frequency units are events/s (Hz).

^2^ mEPSC amplitude units are pico-amperes (pA).

^3^ mEPSC tau decay time constant units are milliseconds (ms).

**Fig 7:**
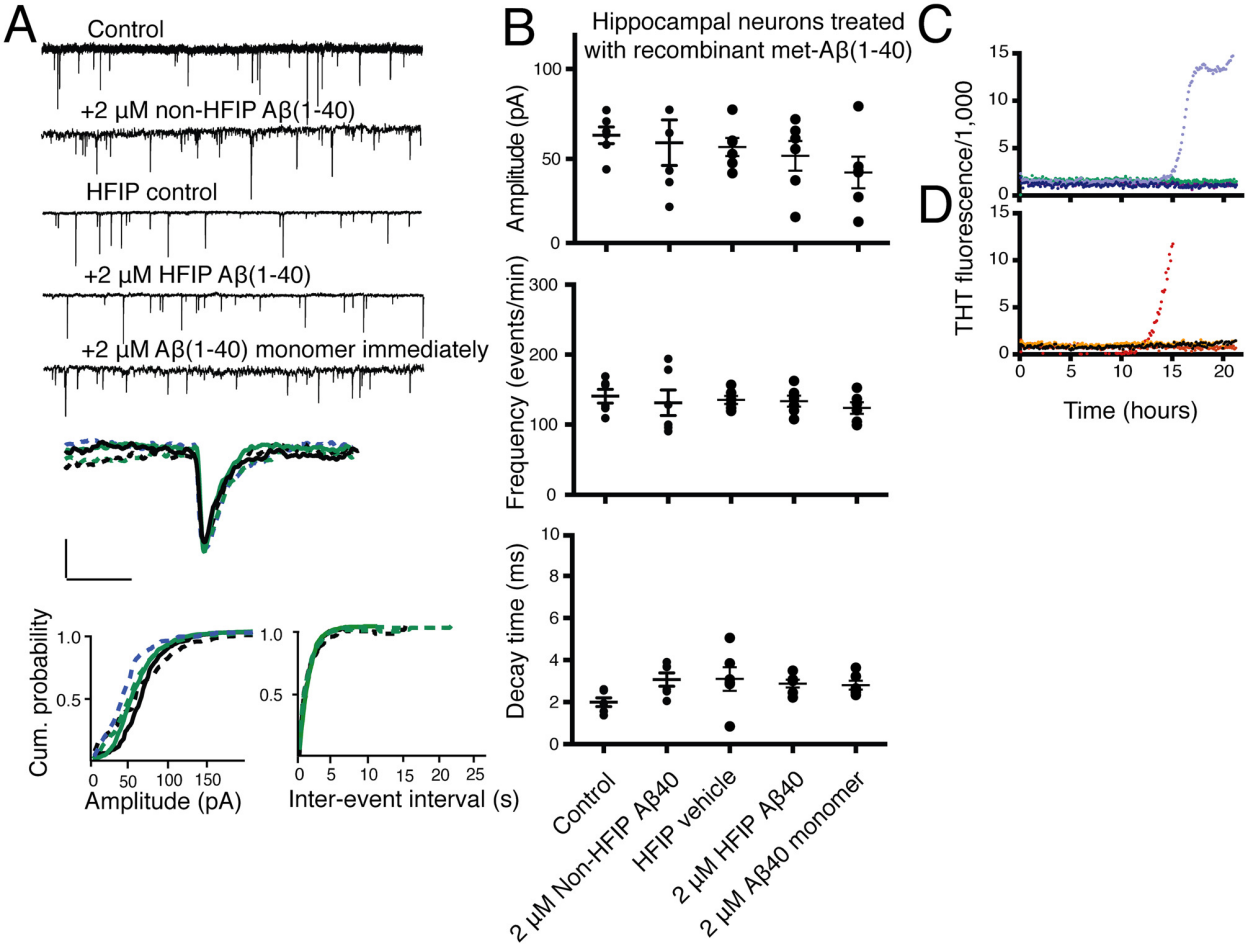
Monomeric recombinant met-Aβ(1–40) does not suppress spontaneous AMPAR-dependent mEPSCs. (A) 2 μM non-HFIP-monomerized met-Aβ(1–40) (dashed black line), 2 μM HFIP-monomerized met-Aβ(1–40) (dashed light green line), and 2 μM direct met-Aβ(1–40) monomer (dashed light blue line) administration do not significantly alter amplitude, frequency, or decay times of AMPAR-dependent mEPSCs. Solid lines, wild type control per condition. The top five tracings are representative tracings of multiple mEPSCs recorded in the conditions indicated above each tracing. The single mEPSC is representative tracing of the average amplitude, rise- and decay-time of recordings from 6 independent experiments, each of which averaged recordings from 3 separate coverslips. Cumulative probabilities represent averages of all 6 experiments. (B) Comparative plots of mEPSCs after 2 μM non-HFIP-monomerized met-Aβ(1–40), 2 μM HFIP-monomerized met-Aβ(1–40), and 2 μM direct met-Aβ(1–40) monomer administration. Kolmogorov-Smirnov test for normality of data distribution. One-way ANOVA, post-hoc Bonferroni. (C) Recombinant met-Aβ(1–40) does not demonstrate aggregation by increased THT fluorescence until 15 hours of incubation under experimental conditions. T_1/2_ for maximal THT fluorescence occurs at 16.1 ± 0.09 hours for 2 μM non-HFIP prepared met-Aβ (1–40) (light purple). 2 μM HFIP-prepared met-Aβ (1–40) (dark purple) does not demonstrate increased THT fluorescence in the experimental window of 22 hours. No 200 nM met-Aβ (1–40) demonstrates fibril formation. 200 nM met-Aβ (1–40), non-HFIP-monomerized (dark blue), HFIP-monomerized (light green). (D) Artificial CSF accelerates monomeric met-Aβ(1–40) aggregation as determined by increased ThT fluorescence but not during the experimental window for neuron treatment. T1/2 to maximal ThT fluorescence is 16.01 ± 0.01 hours for 2 μM met-Aβ(1–40) (red) purified monomer added to neurons immediately following purification. 200 nM met-Aβ(1–40) (orange) in artificial CSF does not demonstrate fibril formation. Purified recombinant met-Aβ(1–40) monomers in sodium-phosphate (NaP) buffer do not exhibit fibril formation, 200 nM (black), 2 μM (yellow).

2 μM recombinant met-Aβ (1–40) preparations, which demonstrated no aggregation, did not affect mEPSCs, indicating that Aβ (1–42) effects were not due to residual monomeric species (Fig 7). These results indicate that presynaptic mEPSC effects are attributable to species present in all Aβ(1–42) preparations but that postsynaptic effects are associated with rapid conformational conversion of Aβ(1–42) during the experimental interval.

**Fig 8:**
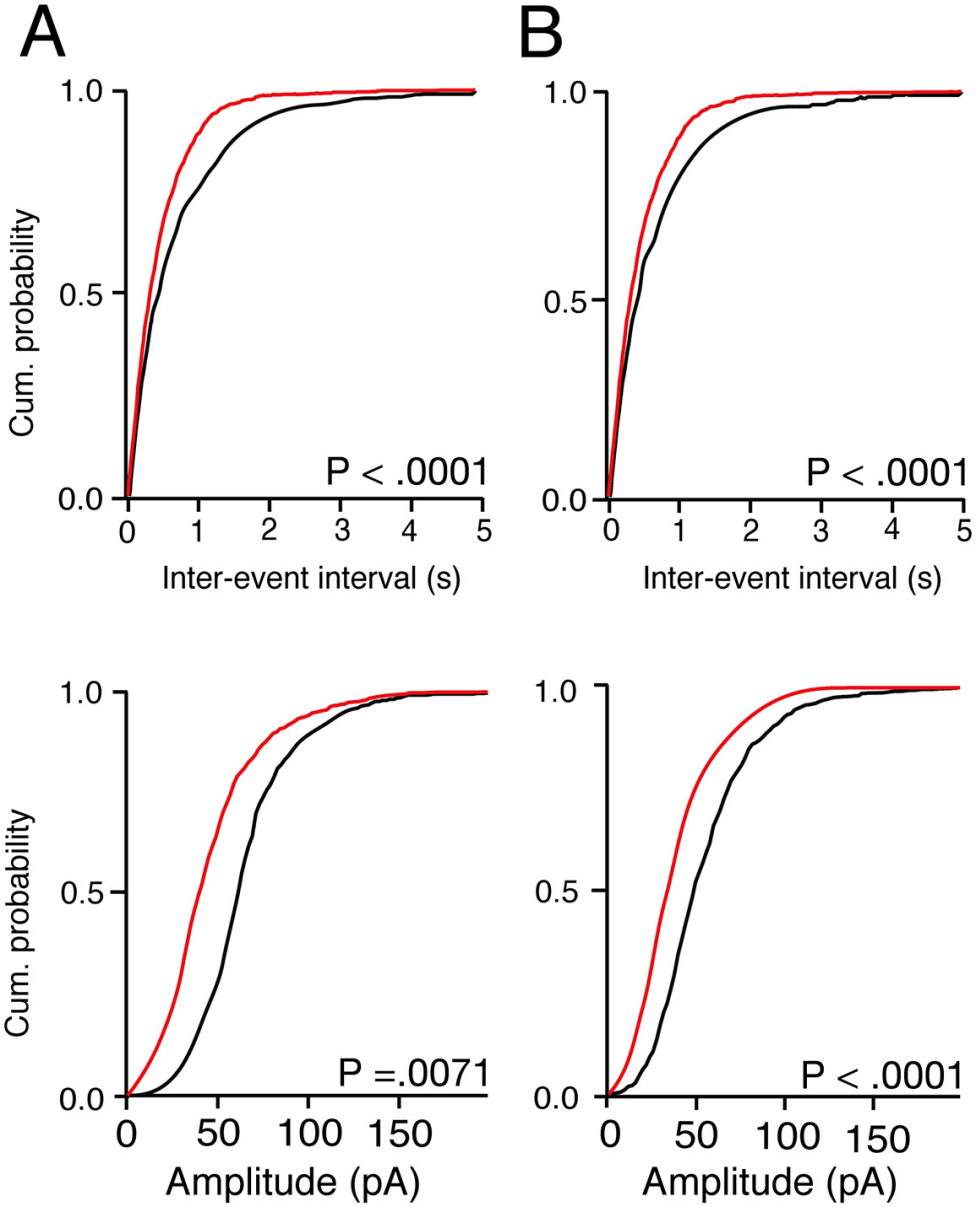
*Cx3cr1* knockout decreases baseline mEPSC amplitude and increases mEPSC frequency in hippocampal neurons. (A) Non-HFIP control aCSF in wild-type and *Cx3cr1* ^*-/-*^ hippocampal neurons. (B) HFIP control artificial CSF in wild-type and *Cx3cr1* ^*-/-*^ hippocampal neurons. Cumulative probabilities represent averages of 6 experiments. Wild-type, black line; *Cx3cr1* ^*-/-*^, red line. See Table 2 for all parameters. Data were inspected for normality of distribution of frequency values by the Kolmogorov-Smirnov test, analyzed for statistical significance by two-way ANOVA by genotype and treatment condition, with post-hoc Bonferroni correction for multiple comparisons.

Because neuronal *Cx3cr1* deficiency inhibits neurotoxicity, we asked if it might also be protective of Aβ (1–42)-induced synaptic dysfunction. Interestingly, we found that relative to wild-type neurons, baseline mEPSC mean frequency and amplitude were significantly increased and decreased in *Cx3cr1* ^*-/-*^ neurons, respectively (Fig 8). The quantile coefficient of variation of quantal amplitude in *Cx3cr1* ^-/-^ hippocampal neurons was significantly greater (.383) than that in wild-type hippocampal neurons (.136; *P* = 0.0001, Mann-Whitney). We also found that no preparation of synthetic (Fig 5C and 5D) or recombinant Aβ(1–42) (Fig 6C and 6D) altered mEPSC frequency, amplitude, or decay time in *Cx3cr1* ^*-/-*^ cultures. See Table 3 for all parameters.

**Table 3:** Aβ-induced mEPSC depression in microglia-depleted mature *Cx3cr1* ^*-/-*^ hippocampal neurons.

Parameter		Prep.	Conc.	Value	SEM
Frequency^1^	Control	Non-HFIP	2 μM	3.73	0.15
Amplitude^2^				43.89	3.42
Decay Time^3^				2.37	.37
Frequency		HFIP	2 μM	2.82	0.23
Amplitude				28.91	5.94
Decay Time				4.79	.99
Frequency	Syn. Aβ (1–42)	Non-HFIP	200 nM	3.29	0.21
Amplitude				37.82	10.59
Decay Time				2.74	.19
Frequency			2 μM	3.47	0.20
Amplitude				34.69	10.93
Decay Time				3.27	.61
Frequency		HFIP	200 nM	2.85	0.12
Amplitude				30.56	11.76
Decay Time				3.28	.32
Frequency			2 μM	2.90	0.33
Amplitude				22.86	4.44
Decay Time				4.28	.65
Frequency	Rec. met-Aβ (1–42)	Non-HFIP	200 nM	3.17	0.43
Amplitude				43.31	11.23
Decay Time				3.64	.93
Frequency			2 μM	3.14	0.39
Amplitude				47.49	15.81
Decay Time				3.40	0.60
Frequency		HFIP	200 nM	3.22	0.31
Amplitude				40.80	3.90
Decay Time				5.48	1.25
Frequency			2 μM	3.48	0.46
Amplitude				23.82	5.86
Decay Time				5.08	1.28

^1^ mEPSC frequency units are events/s (Hz).

^2^ mEPSC amplitude units are pico-amperes (pA).

^3^ mEPSC tau decay time constant units are milliseconds (ms).

**Fig 9:**
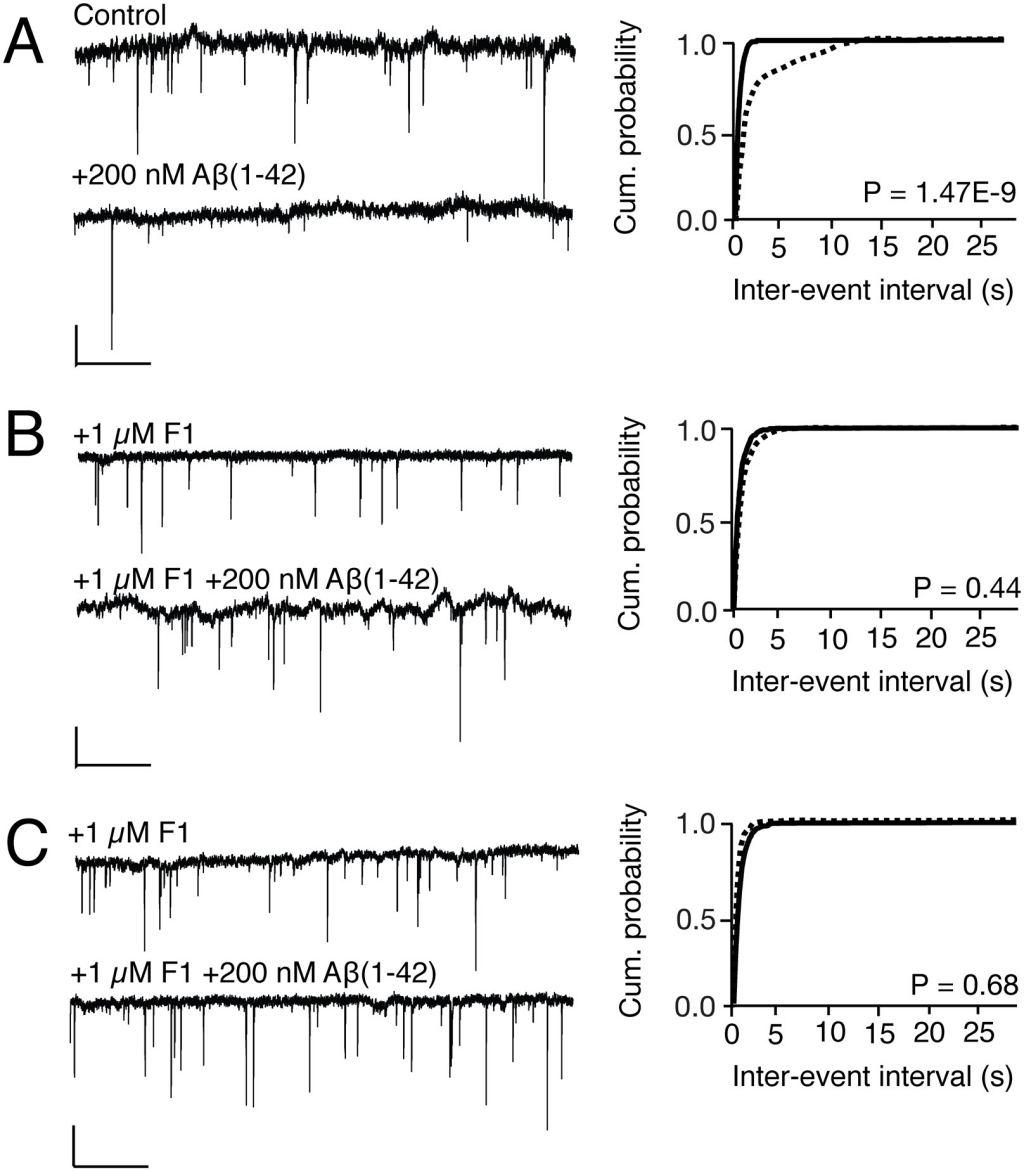
Acute and chronic antagonism of neuronal Cx3cr1 precludes Aβ(1–42) suppression of spontaneous AMPAR-dependent mEPSCs in wild-type neurons. (A) 200 nM non-HFIP monomerized Aβ(1–42) in wild-type neurons (61.64 ± 9.09 events/min vs. 176.64 ± 18.97 events/min, control, *P* = .001, Kruskal-Wallis, post-hoc multiple comparisons). (B) 200 nM non-HFIP monomerized Aβ(1–42) in wild-type neurons pre-treated acutely with 1 μM amino terminus modified CX3CR1 ligand F1 (128.92 events/min ± 19.73 vs. 157.35 events/min ± 30.67, acute F1-treated control). (C) 200 nM non-HFIP monomerized Aβ(1–42) in wild-type neurons pre-treated chronically with 1 μM amino terminus modified CX3CR1 ligand F1 (205.21 events/min ± 15.63 vs. 196.84 events/min ± 15.77, chronic F1-treated control). The left six tracings indicate multiple mEPSCs, representative of recordings from nine individual coverslips in each condition indicated above each tracing. Cumulative probabilities represent averages of all nine coverslips in each condition. Dashed lines represent Aβ-treated condition and solid lines indicate vehicle-treated controls in each pair. Kolmogorov-Smirnov for differences in cumulative distributions, post-hoc Bonferroni. One-way ANOVA for differences in mean frequencies.

To further investigate whether functional neuronal Cx3cr1 facilitates Aβ(1–42)-induced suppression of AMPA-mediated mEPSCs, we treated wild-type hippocampal culture with the modified amino terminus Cx3Cl1 ligand, F1, which is a Cx3cr1 antagonist, either concurrently with (acute) or on DIV3 preceding treatment with (chronic) 200 nM non-HFIP Aβ (1–42). We found that both acute and chronic treatment with 1 μM F1 blocked Aβ (1–42)-induced reduction in AMPA-mediated mEPSC frequency and rightward shifting of the cumulative frequency distribution curve of inter event intervals (Fig 9). Thus, genetic inactivation, as well as acute and chronic pharmacologic blockade of Cx3cr1 protect against Aβ-induced synaptotoxicity.

## Discussion

The three principal results of this study are: (1) neuronal *Cx3cr1* deficiency abates Aβ (1–42)-induced cytotoxicity; (2) distinct conformational species of Aβ (1–42) affect spontaneous synaptic transmission differentially; and (3) *Cx3cr1* deficiency protects cultured mouse hippocampal neurons from differential Aβ (1–42)-induced defects in spontaneous synaptic transmission. Taken together our data suggest that Cx3cr1 promotes AD-like pathology by promoting neurotoxic and synaptotoxic effects of Aβ (1–42).

Our results suggest that neuronal *Cx3cr1* deficiency may play a neuroprotective role in the context of Aβ toxicity, but previous studies have been inconsistent with regards to neuronal expression of the receptor [4, 11]. Regional specificity of neuronal Cx3cr1 expression might be one reason for these conflicting reports. In our experiments, we found that both cultured mixed cortical and hippocampal neurons expressed *Cx3cr1* mRNA but that receptor protein was expressed more robustly in hippocampal neurons, in accordance with previous reports of selective hippocampal CA1 neuron marker colocalization with Cx3cr1 [14].

Our results are consistent with previous reports that hippocampal neurons are more susceptible than cortical neurons to Aβ toxicity [48]. Because Cx3cr1 expression is required for Aβ toxicity in neuronal culture, selective expression of Cx3cr1 in hippocampal neurons might contribute to their heightened susceptibility to Aβ. Discrepancies in reports of neuronal Cx3cr1 expression [11–16] might also be a result of developmental and inducible receptor regulation. All reports of cultured neuronal Cx3cr1 expression occur at DIV7 or later, and several excitotoxic stimuli, including Aβ, elevate Cx3cl1-Cx3cr1 signaling [12, 16]. Interestingly, studies conducted within the human central nervous system report neuronal Cx3cr1 expression [13, 16].

Dissociated, *Cx3cr1* ^*-/-*^ microglia and neurons exhibited opposite cytotoxic responses to Aβ, indicating different receptor effector functions. Whether Cx3cl1 signals through microglial or neuronal Cx3cr1 may depend on relative receptor affinities for the ligand, a suggestion supported by reports of microglial activation by low concentrations of Cx3cl1 and direct neuronal activation by higher concentrations [12, 19]. Our study suggests that cell-specificity of receptor expression may be an important consideration when developing therapeutics targeting the Cx3cl1-Cx3cr1 signaling axis and other chemokine receptors expressed by both neurons and glial cells in the CNS.

In our study, Aβ increased soluble Cx3cl1 from cultured hippocampal neurons. Previous studies have implicated Cx3cl1 in concentration-dependent reduction of neurotransmitter release probability [32], reduction of amplitude of evoked AMPA currents, and direct AMPAR internalization [12, 19, 31, 32], which are downstream effects of Aβ. Potentiation of Cx3cl1 signalling through neuronal Cx3cr1, therefore, might be one mechanism of Aβ toxicity. These *in vitro* results warrant validation *in vivo*.

We found that Aβ(1–42) selectively depressed pre- or both pre- and postsynaptic parameters of AMPAR-dependent mEPSCs depending on peptide aggregation-state and that *Cx3cr1* was required for both effects of Aβ. It should be noted that while mEPSC frequency is considered to be primarily a presynaptic measure of release probability, we cannot rule out potential postsynaptic mechanisms that might contribute to frequency alterations. Further, due to decreased baseline mEPSC amplitude in *Cx3cr1* ^*-/-*^ neurons, we cannot rule out inability to detect Aβ-induced amplitude reduction in these cells. While concentration-dependent selective Aβ reduction of mEPSC frequency has been reported previously [30], the effect we observed was not simply concentration-dependent, since only 2 μM Aβ preparations that also demonstrated rapid sigmoidal kinetics of aggregation during the recording interval induced postsynaptic effects. Collectively, the kinetic and AFM data suggest that metastable conformations associated with fibril formation were required to affect both pre- and postsynaptic parameters of mEPSCs in hippocampal culture. This finding is supported by previous studies indicating that severity and mechanisms of Aβ toxicity are known to vary, dependent on peptide aggregation state [49, 52–54]. It should be noted that neurons treated with HFIP Aβ demonstrated slightly decreased baseline frequency and amplitude, with more pronounced effects in *Cx3cr1* ^*-/-*^ neurons, corroborating previous reports that residual solvent may be toxic [55]. Residual solvent may also contribute to slowed aggregation kinetics of HFIP Aβ(1–42).

2 μM non-HFIP synthetic and recombinant Aβ(1–42) demonstrated comparatively high baseline ThT fluorescence and impeded aggregation profiles relative to model sigmoidal kinetics curves; as assessed by AFM, both preparations contained pre-existent fibrils, which likely contributed to these aggregation profiles. However, the ability of Aβ to affect postsynaptic mEPSC parameters did not require these pre-existent fibrils as non-fibril containing 2 μM HFIP met-Aβ (1–42) elicited similar mEPSC amplitude reduction.

There are several limitations to the application of this study’s results to pathophysiologic mechanisms of the aging human CNS, owing to its *in vitro* nature, its dependence on neonatal murine tissue, and the requirement for doses of Aβ that exceed physiological concentrations in humans to elicit neurotoxicity in this system.

In conclusion, our study shows that Aβ (1–42) intermediates present in all preparations decrease presynaptic release probability, while metastable conformations populated during the rapid phase of sigmoidal aggregation involve additional postsynaptic mechanisms of receptor internalization and desensitization. Because *Cx3cr1* deficiency conferred resistance to both presynaptic and postsynaptic mEPSC parameter depression and because baseline mEPSC frequency and amplitude, were increased and decreased, respectively, in *Cx3cr1* knockout neurons when compared to wild-type neurons, the neuroprotective effects of receptor knockout could be related to global network alterations or to specific receptor-Aβ interactions.

Long-term modulation of synaptic scaling by AMPAR stabilization has been suggested as a viable disease-modifying strategy in AD [56]. Our study implicates neuronal Cx3cr1 in network modifications that alter susceptibility to Aβ-induced mEPSC depression, indicating Cx3cr1 as a potential therapeutic target.

## References

[1] Selkoe DJ. Alzheimer's disease is a synaptic failure. Science. 2002;298(5594):789–91. Epub 2002/10/26. 10.1126/science.1074069 . 12399581

[2] Miyata S, Nishimura Y, Nakashima T. Perineuronal nets protect against amyloid beta-protein neurotoxicity in cultured cortical neurons. Brain Res. 2007;1150:200–6. Epub 2007/04/03. 10.1016/j.brainres.2007.02.066 . 17397805

[3] Perry VH, Nicoll JA, Holmes C. Microglia in neurodegenerative disease. Nat Rev Neurol. 2010;6(4):193–201. Epub 2010/03/18. 10.1038/nrneurol.2010.17 . 20234358

[4] Fuhrmann M, Bittner T, Jung CK, Burgold S, Page RM, Mitteregger G, et al Microglial Cx3cr1 knockout prevents neuron loss in a mouse model of Alzheimer's disease. Nat Neurosci. 2010;13(4):411–3. Epub 2010/03/23. 10.1038/nn.2511 . 20305648 PMC4072212

[5] Lee S, Varvel NH, Konerth ME, Xu G, Cardona AE, Ransohoff RM, et al CX3CR1 deficiency alters microglial activation and reduces beta-amyloid deposition in two Alzheimer's disease mouse models. Am J Pathol. 2010;177(5):2549–62. Epub 2010/09/25. 10.2353/ajpath.2010.100265 ; PubMed Central PMCID: PMCPmc2966811. 20864679 PMC2966811

[6] Liu Z, Condello C, Schain A, Harb R, Grutzendler J. CX3CR1 in microglia regulates brain amyloid deposition through selective protofibrillar amyloid-beta phagocytosis. J Neurosci. 2010;30(50):17091–101. Epub 2010/12/17. 10.1523/jneurosci.4403-10.2010 ; PubMed Central PMCID: PMCPmc3077120. 21159979 PMC3077120

[7] Wu J, Bie B, Yang H, Xu JJ, Brown DL, Naguib M. Suppression of central chemokine fractalkine receptor signaling alleviates amyloid-induced memory deficiency. Neurobiol Aging. 2013;34(12):2843–52. Epub 2013/07/17. 10.1016/j.neurobiolaging.2013.06.003 . 23855980

[8] Cho SH, Sun B, Zhou Y, Kauppinen TM, Halabisky B, Wes P, et al CX3CR1 protein signaling modulates microglial activation and protects against plaque-independent cognitive deficits in a mouse model of Alzheimer disease. J Biol Chem. 2011;286(37):32713–22. Epub 2011/07/21. 10.1074/jbc.M111.254268 ; PubMed Central PMCID: PMCPmc3173153. 21771791 PMC3173153

[9] Rogers JT, Morganti JM, Bachstetter AD, Hudson CE, Peters MM, Grimmig BA, et al CX3CR1 deficiency leads to impairment of hippocampal cognitive function and synaptic plasticity. J Neurosci. 2011;31(45):16241–50. Epub 2011/11/11. 10.1523/jneurosci.3667-11.2011 ; PubMed Central PMCID: PMCPmc3236509. 22072675 PMC3236509

[10] Hebron ML, Algarzae NK, Lonskaya I, Moussa C. Fractalkine signaling and Tau hyper-phosphorylation are associated with autophagic alterations in lentiviral Tau and Abeta(1–42) gene transfer models. Exp Neurol. 2013 Epub 2013/01/22. 10.1016/j.expneurol.2013.01.009 ; PubMed Central PMCID: PMCPmc3644355. 23333589 PMC3644355

[11] Ransohoff RM, Liu L, Cardona AE. Chemokines and chemokine receptors: multipurpose players in neuroinflammation. Int Rev Neurobiol. 2007;82:187–204. Epub 2007/08/07. 10.1016/s0074-7742(07)82010-1 . 17678962

[12] Meucci O, Fatatis A, Simen AA, Bushell TJ, Gray PW, Miller RJ. Chemokines regulate hippocampal neuronal signaling and gp120 neurotoxicity. Proc Natl Acad Sci U S A. 1998;95(24):14500–5. Epub 1998/11/25. ; PubMed Central PMCID: PMCPmc24402. 9826729 10.1073/pnas.95.24.14500 PMC24402

[13] Tong N, Perry SW, Zhang Q, James HJ, Guo H, Brooks A, et al Neuronal fractalkine expression in HIV-1 encephalitis: roles for macrophage recruitment and neuroprotection in the central nervous system. J Immunol. 2000;164(3):1333–9. Epub 2000/01/21. . 10640747 10.4049/jimmunol.164.3.1333

[14] Hughes PM, Botham MS, Frentzel S, Mir A, Perry VH. Expression of fractalkine (CX3CL1) and its receptor, CX3CR1, during acute and chronic inflammation in the rodent CNS. Glia. 2002;37(4):314–27. Epub 2002/03/01. . 11870871

[15] Hatori K, Nagai A, Heisel R, Ryu JK, Kim SU. Fractalkine and fractalkine receptors in human neurons and glial cells. J Neurosci Res. 2002;69(3):418–26. Epub 2002/07/19. 10.1002/jnr.10304 . 12125082

[16] Gillard SE, Lu M, Mastracci RM, Miller RJ. Expression of functional chemokine receptors by rat cerebellar neurons. J Neuroimmunol. 2002;124(1–2):16–28. Epub 2002/04/18. . 11958818 10.1016/s0165-5728(02)00005-x

[17] Mizuno T, Kawanokuchi J, Numata K, Suzumura A. Production and neuroprotective functions of fractalkine in the central nervous system. Brain Res. 2003;979(1–2):65–70. Epub 2003/07/10. . 12850572 10.1016/s0006-8993(03)02867-1

[18] Deiva K, Geeraerts T, Salim H, Leclerc P, Hery C, Hugel B, et al Fractalkine reduces N-methyl-d-aspartate-induced calcium flux and apoptosis in human neurons through extracellular signal-regulated kinase activation. Eur J Neurosci. 2004;20(12):3222–32. Epub 2004/12/22. 10.1111/j.1460-9568.2004.03800.x . 15610155

[19] Limatola C, Lauro C, Catalano M, Ciotti MT, Bertollini C, Di Angelantonio S, et al Chemokine CX3CL1 protects rat hippocampal neurons against glutamate-mediated excitotoxicity. J Neuroimmunol. 2005;166(1–2):19–28. Epub 2005/07/16. 10.1016/j.jneuroim.2005.03.023 . 16019082

[20] Shan S, Hong-Min T, Yi F, Jun-Peng G, Yue F, Yan-Hong T, et al New evidences for fractalkine/CX3CL1 involved in substantia nigral microglial activation and behavioral changes in a rat model of Parkinson's disease. Neurobiol Aging. 2011;32(3):443–58. Epub 2009/04/17. 10.1016/j.neurobiolaging.2009.03.004 . 19368990

[21] Chapman GA, Moores K, Harrison D, Campbell CA, Stewart BR, Strijbos PJ. Fractalkine cleavage from neuronal membranes represents an acute event in the inflammatory response to excitotoxic brain damage. J Neurosci. 2000;20(15):Rc87 Epub 2000/07/19. . 10899174 10.1523/JNEUROSCI.20-15-j0004.2000 PMC6772533

[22] Wang J, Ohno-Matsui K, Nakahama K, Okamoto A, Yoshida T, Shimada N, et al Amyloid beta enhances migration of endothelial progenitor cells by upregulating CX3CR1 in response to fractalkine, which may be associated with development of choroidal neovascularization. Arterioscler Thromb Vasc Biol. 2011;31(7):e11–8. Epub 2011/04/30. 10.1161/atvbaha.110.215517 . 21527754

[23] Shankar GM, Walsh DM. Alzheimer's disease: synaptic dysfunction and Abeta. Mol Neurodegener. 2009;4:48 Epub 2009/11/26. 10.1186/1750-1326-4-48 ; PubMed Central PMCID: PMCPmc2788538. 19930651 PMC2788538

[24] Small DH, Mok SS, Bornstein JC. Alzheimer's disease and Abeta toxicity: from top to bottom. Nat Rev Neurosci. 2001;2(8):595–8. Epub 2001/08/03. 10.1038/35086072 . 11484003

[25] Chang EH, Savage MJ, Flood DG, Thomas JM, Levy RB, Mahadomrongkul V, et al AMPA receptor downscaling at the onset of Alzheimer's disease pathology in double knockin mice. Proc Natl Acad Sci U S A. 2006;103(9):3410–5. Epub 2006/02/24. 10.1073/pnas.0507313103 ; PubMed Central PMCID: PMCPmc1413872. 16492745 PMC1413872

[26] Hsieh H, Boehm J, Sato C, Iwatsubo T, Tomita T, Sisodia S, et al AMPAR removal underlies Abeta-induced synaptic depression and dendritic spine loss. Neuron. 2006;52(5):831–43. Epub 2006/12/06. 10.1016/j.neuron.2006.10.035 ; PubMed Central PMCID: PMCPmc1850952. 17145504 PMC1850952

[27] Parameshwaran K, Dhanasekaran M, Suppiramaniam V. Amyloid beta peptides and glutamatergic synaptic dysregulation. Exp Neurol. 2008;210(1):7–13. Epub 2007/12/07. 10.1016/j.expneurol.2007.10.008 . 18053990

[28] Gu Z, Liu W, Yan Z. {beta}-Amyloid impairs AMPA receptor trafficking and function by reducing Ca2+/calmodulin-dependent protein kinase II synaptic distribution. J Biol Chem. 2009;284(16):10639–49. Epub 2009/02/26. 10.1074/jbc.M806508200 ; PubMed Central PMCID: PMCPmc2667751. 19240035 PMC2667751

[29] Parodi J, Sepulveda FJ, Roa J, Opazo C, Inestrosa NC, Aguayo LG. Beta-amyloid causes depletion of synaptic vesicles leading to neurotransmission failure. J Biol Chem. 2010;285(4):2506–14. Epub 2009/11/17. 10.1074/jbc.M109.030023 ; PubMed Central PMCID: PMCPmc2807307. 19915004 PMC2807307

[30] Zhao WQ, Santini F, Breese R, Ross D, Zhang XD, Stone DJ, et al Inhibition of calcineurin-mediated endocytosis and alpha-amino-3-hydroxy-5-methyl-4-isoxazolepropionic acid (AMPA) receptors prevents amyloid beta oligomer-induced synaptic disruption. J Biol Chem. 2010;285(10):7619–32. Epub 2009/12/25. 10.1074/jbc.M109.057182 ; PubMed Central PMCID: PMCPmc2844209. 20032460 PMC2844209

[31] Ragozzino D, Di Angelantonio S, Trettel F, Bertollini C, Maggi L, Gross C, et al Chemokine fractalkine/CX3CL1 negatively modulates active glutamatergic synapses in rat hippocampal neurons. J Neurosci. 2006;26(41):10488–98. Epub 2006/10/13. 10.1523/jneurosci.3192-06.2006 . 17035533 PMC6674698

[32] Piccinin S, Di Angelantonio S, Piccioni A, Volpini R, Cristalli G, Fredholm BB, et al CX3CL1-induced modulation at CA1 synapses reveals multiple mechanisms of EPSC modulation involving adenosine receptor subtypes. J Neuroimmunol. 2010;224(1–2):85–92. Epub 2010/06/24. 10.1016/j.jneuroim.2010.05.012 . 20570369

[33] Walsh DM, Selkoe DJ. A beta oligomers—a decade of discovery. J Neurochem. 2007;101(5):1172–84. Epub 2007/02/09. 10.1111/j.1471-4159.2006.04426.x . 17286590

[34] Shankar GM, Li S, Mehta TH, Garcia-Munoz A, Shepardson NE, Smith I, et al Amyloid-beta protein dimers isolated directly from Alzheimer's brains impair synaptic plasticity and memory. Nat Med. 2008;14(8):837–42. Epub 2008/06/24. 10.1038/nm1782 ; PubMed Central PMCID: PMCPmc2772133. 18568035 PMC2772133

[35] Nimmrich V, Grimm C, Draguhn A, Barghorn S, Lehmann A, Schoemaker H, et al Amyloid beta oligomers (A beta(1–42) globulomer) suppress spontaneous synaptic activity by inhibition of P/Q-type calcium currents. J Neurosci. 2008;28(4):788–97. Epub 2008/01/25. 10.1523/jneurosci.4771-07.2008 . 18216187 PMC6671006

[36] Roselli F, Tirard M, Lu J, Hutzler P, Lamberti P, Livrea P, et al Soluble beta-amyloid1-40 induces NMDA-dependent degradation of postsynaptic density-95 at glutamatergic synapses. J Neurosci. 2005;25(48):11061–70. Epub 2005/12/02. 10.1523/jneurosci.3034-05.2005 . 16319306 PMC6725651

[37] Almeida CG, Tampellini D, Takahashi RH, Greengard P, Lin MT, Snyder EM, et al Beta-amyloid accumulation in APP mutant neurons reduces PSD-95 and GluR1 in synapses. Neurobiol Dis. 2005;20(2):187–98. Epub 2005/10/26. 10.1016/j.nbd.2005.02.008 . 16242627

[38] Knowles TP, Waudby CA, Devlin GL, Cohen SI, Aguzzi A, Vendruscolo M, et al An analytical solution to the kinetics of breakable filament assembly. Science. 2009;326(5959):1533–7. Epub 2009/12/17. 10.1126/science.1178250 . 20007899

[39] Cohen SI, Vendruscolo M, Welland ME, Dobson CM, Terentjev EM, Knowles TP. Nucleated polymerization with secondary pathways. I. Time evolution of the principal moments. J Chem Phys. 2011;135(6):065105 Epub 2011/08/17. 10.1063/1.3608916 . 21842954 PMC5017532

[40] Shipton OA, Leitz JR, Dworzak J, Acton CE, Tunbridge EM, Denk F, et al Tau protein is required for amyloid {beta}-induced impairment of hippocampal long-term potentiation. J Neurosci. 2011;31(5):1688–92. Epub 2011/02/04. 10.1523/jneurosci.2610-10.2011 . 21289177 PMC3836238

[41] Walsh DM, Klyubin I, Fadeeva JV, Cullen WK, Anwyl R, Wolfe MS, et al Naturally secreted oligomers of amyloid beta protein potently inhibit hippocampal long-term potentiation in vivo. Nature. 2002;416(6880):535–9. Epub 2002/04/05. 10.1038/416535a . 11932745

[42] Fa M, Orozco IJ, Francis YI, Saeed F, Gong Y, Arancio O. Preparation of oligomeric beta-amyloid 1–42 and induction of synaptic plasticity impairment on hippocampal slices. J Vis Exp. 2010;(41). Epub 2010/07/21. 10.3791/1884 ; PubMed Central PMCID: PMCPmc3156071. 20644518 PMC3156071

[43] Walsh DM, Thulin E, Minogue AM, Gustavsson N, Pang E, Teplow DB, et al A facile method for expression and purification of the Alzheimer's disease-associated amyloid beta-peptide. Febs j. 2009;276(5):1266–81. Epub 2009/01/30. 10.1111/j.1742-4658.2008.06862.x ; PubMed Central PMCID: PMCPmc2702495. 19175671 PMC2702495

[44] Dorgham K, Ghadiri A, Hermand P, Rodero M, Poupel L, Iga M, et al An engineered CX3CR1 antagonist endowed with anti-inflammatory activity. J Leukoc Biol. 2009;86(4):903–11. Epub 2009/07/03. 10.1189/jlb.0308158 . 19571253

[45] Bhaskar K, Konerth M, Kokiko-Cochran ON, Cardona A, Ransohoff RM, Lamb BT. Regulation of tau pathology by the microglial fractalkine receptor. Neuron. 2010;68(1):19–31. Epub 2010/10/06. 10.1016/j.neuron.2010.08.023 ; PubMed Central PMCID: PMCPmc2950825. 20920788 PMC2950825

[46] Liang JH, Du J, Xu LD, Jiang T, Hao S, Bi J, et al Catalpol protects primary cultured cortical neurons induced by Abeta(1–42) through a mitochondrial-dependent caspase pathway. Neurochem Int. 2009;55(8):741–6. Epub 2009/07/28. 10.1016/j.neuint.2009.07.004 . 19631247

[47] Parameshwaran K, Sims C, Kanju P, Vaithianathan T, Shonesy BC, Dhanasekaran M, et al Amyloid beta-peptide Abeta(1–42) but not Abeta(1–40) attenuates synaptic AMPA receptor function. Synapse. 2007;61(6):367–74. Epub 2007/03/21. 10.1002/syn.20386 . 17372971

[48] Lambert MP, Barlow AK, Chromy BA, Edwards C, Freed R, Liosatos M, et al Diffusible, nonfibrillar ligands derived from Abeta1-42 are potent central nervous system neurotoxins. Proc Natl Acad Sci U S A. 1998;95(11):6448–53. Epub 1998/05/30. ; PubMed Central PMCID: PMCPmc27787. 9600986 10.1073/pnas.95.11.6448 PMC27787

[49] Bernstein SL, Dupuis NF, Lazo ND, Wyttenbach T, Condron MM, Bitan G, et al Amyloid-beta protein oligomerization and the importance of tetramers and dodecamers in the aetiology of Alzheimer's disease. Nat Chem. 2009;1(4):326–31. Epub 2010/08/13. 10.1038/nchem.247 ; PubMed Central PMCID: PMCPmc2918915. 20703363 PMC2918915

[50] Raymond CR, Ireland DR, Abraham WC. NMDA receptor regulation by amyloid-beta does not account for its inhibition of LTP in rat hippocampus. Brain Res. 2003;968(2):263–72. Epub 2003/03/29. . 12663096 10.1016/s0006-8993(03)02269-8

[51] Zagorski MG, Yang J, Shao H, Ma K, Zeng H, Hong A. Methodological and chemical factors affecting amyloid beta peptide amyloidogenicity. Methods Enzymol. 1999;309:189–204. Epub 1999/10/03. . 10507025 10.1016/s0076-6879(99)09015-1

[52] Deshpande A, Mina E, Glabe C, Busciglio J. Different conformations of amyloid beta induce neurotoxicity by distinct mechanisms in human cortical neurons. J Neurosci. 2006;26(22):6011–8. Epub 2006/06/02. 10.1523/jneurosci.1189-06.2006 . 16738244 PMC6675207

[53] Ono K, Condron MM, Teplow DB. Structure-neurotoxicity relationships of amyloid beta-protein oligomers. Proc Natl Acad Sci U S A. 2009;106(35):14745–50. Epub 2009/08/27. 10.1073/pnas.0905127106 ; PubMed Central PMCID: PMCPmc2736424. 19706468 PMC2736424

[54] Cizas P, Budvytyte R, Morkuniene R, Moldovan R, Broccio M, Losche M, et al Size-dependent neurotoxicity of beta-amyloid oligomers. Arch Biochem Biophys. 2010;496(2):84–92. Epub 2010/02/16. 10.1016/j.abb.2010.02.001 ; PubMed Central PMCID: PMCPmc2853175. 20153288 PMC2853175

[55] Capone R, Quiroz FG, Prangkio P, Saluja I, Sauer AM, Bautista MR, et al Amyloid-beta-induced ion flux in artificial lipid bilayers and neuronal cells: resolving a controversy. Neurotox Res. 2009;16(1):1–13. Epub 2009/06/16. 10.1007/s12640-009-9033-1 ; PubMed Central PMCID: PMCPmc2864106. 19526294 PMC2864106

[56] Lynch G. Memory enhancement: the search for mechanism-based drugs. Nat Neurosci. 2002;5 Suppl:1035–8. Epub 2002/10/31. 10.1038/nn935 . 12403980

